# A modular genetic toolbox for precise gene regulation and multi-color imaging in streptococci

**DOI:** 10.1093/femsml/uqag006

**Published:** 2026-02-26

**Authors:** Johann Mignolet, Albane Schmid, Johan Staub, Jan Roelof van der Meer, Jan-Willem Veening, Virginie Libante

**Affiliations:** Department of Fundamental Microbiology, Faculty of Biology and Medicine, University of Lausanne, CH-1015 Lausanne, Switzerland; Department of Fundamental Microbiology, Faculty of Biology and Medicine, University of Lausanne, CH-1015 Lausanne, Switzerland; Université de Lorraine, INRAE, DynAMic, F-54000 Nancy, France; Department of Fundamental Microbiology, Faculty of Biology and Medicine, University of Lausanne, CH-1015 Lausanne, Switzerland; Department of Fundamental Microbiology, Faculty of Biology and Medicine, University of Lausanne, CH-1015 Lausanne, Switzerland; Université de Lorraine, INRAE, DynAMic, F-54000 Nancy, France

**Keywords:** streptococcus, subcellular localization, HlpA, HU, multiple fluorescent labeling, inducible promoter

## Abstract

Fluorescent labeling is a powerful tool in microbiology allowing live cell imaging and providing insights into dynamic cellular processes, quantification of gene expression, and protein subcellular localization. Although multicolor imaging is widely used in *Streptococcus pneumoniae* and *S. mutans*, this is less common in other streptococcal species. To address this gap in the streptococcal molecular toolbox, we benchmarked five different fluorescent proteins. They were fused to the C-terminus of *S. pneumoniae* HlpA, a small non-specific DNA binding histone-like protein. These reporters, combined with four different antibiotic resistance genes, were engineered with various expression systems (inducible or constitutive) to form versatile cassettes. We provide methods to transfer these cassettes to different streptococcal species including *S. salivarius, S. thermophilus*, and *S. pyogenes*. As a proof of concept, we generated a triple labeled *S. salivarius* strain in which HlpA, FtsZ, and DivIVA were fused to three spectrally distinct compatible fluorescent proteins. Multiple fluorescent labeling has broad applications for deciphering a wide range of scientific problems, from cellular processes to infectious disease mechanisms. The availability of these cassettes should allow for a wider use of single-cell labeling strategies in the streptococcus clade and other closely related bacteria.

## Teaser

New genetic and fluorescent tools in lactic acid bacteria to monitor populations and protein dynamics at the single cell level.

## Introduction

Since the mid-1990s, the use of the green fluorescent protein (GFP) has been a powerful biomolecular tool for gene expression, protein localization/tracking, and live cell detection (Chalfie 1995, Southward and Surette 2002). In bacteria, starting with *Escherichia coli, Bacillus subtilis*, and *Pseudomonas putida*, GFP has been successfully used to study protein and DNA localization (Webb et al. 1995, Sorensen et al. 2003). GFP variants have been selected to solve problems of low intensity, folding, solubility, and inclusion bodies at elevated temperatures (Phillips 2001). In parallel, mutant proteins with different excitation and emission spectra and fluorescence intensity were discovered or engineered to allow for multiple dynamic labeling of bacterial communities (Errampalli et al. 1999), or proteins (Gitai et al. 2005).

The human pathogen *Streptococcus pneumoniae* is a Gram-positive bacterial species for which many molecular tools have been developed. Extensive work has been done on *S. pneumoniae* to develop labeling strategies, and a collection of compatible fluorescent proteins (FPs) for multiple and dynamic labeling optimized for super-resolution microscopy have been made available (listed in Keller et al. 2019 and Kjos 2019). The benefits of multi-labeling could be two-fold. It allows to (i) discriminate between several populations of cells/species labeled with compatible fluorescent proteins [for instance with *Streptococcus mutans* (Shields et al. 2019, Peters et al. 2025)], or (ii) simultaneously track the subcellular location of numerous proteins in a single cell, as shown with integrative vectors (Keller et al. 2019). Besides these numerous studies, several papers reported the implementation of labeling (codon optimization-based) strategies in other streptococcal species (Knoops et al. 2022a, Lun and Willson 2004, Chen et al. 2012, Dutton et al. 2014, Vickerman et al. 2015, Reck and Wagner-Dobler 2016, Sullivan and Ulett 2018, Liang et al. 2019, Anandan et al. 2024, Lautenschlager et al. 2024).

Sufficient and adequate abundance of FPs is a prerequisite for efficient population or cell monitoring. To improve expression of fluorescent proteins, translational fusions to highly abundant and stable proteins have been engineered (Chalfie 1995). In *S. pneumoniae*, the histone-like protein HlpA (a.k.a. HU) fused to an FP at its C-terminal end, gave remarkably brightly homogeneously labeled cells (Kjos et al. 2015). This was due to (i) the stable chromosomal integration of the fusion gene downstream of native *hlpA*, (ii) the high transcript levels of *hlpA* throughout all stages of the cell cycle, and (iii) the nucleoid association of the fluorescent signal, without deleterious effects, which limits the diffusion of the FP. This approach was first applied to *S. pneumoniae* for *in vivo* live cell imaging of two different HlpA-FP labeled strains within mouse infection experiments (Kjos et al. 2015) and then, for biofilm formation with two combined strains (Valente et al. 2021). HlpA is essential for cell survival in streptococci (Liu et al. 2008, Bugrysheva et al. 2011, Biswas and Mohapatra 2012), and is largely conserved across prokaryotes (Stinson et al. 1998, Ferrandiz et al. 2018), allowing this labeling strategy to be applied to a wider range of bacteria.

In parallel to protein stability as such, the system and timing of expression is of major importance. Constitutive production of (recombinant) proteins can have drastic effects on cell physiology, slowing down growth rate or provoking abnormal cell morphologies (Kintaka et al. 2020, James et al. 2021). The overload of overproduced protein can consume the energy and nutrients of a cell and influence its metabolic state. Moreover, inappropriate timing of expression can collide with the cell cycle. Inducible expression systems offer a solution to minimize collateral toxicity from protein overproduction. Most studied promoters from lactic acid bacteria are leaky, trigger too strong expression or are not sufficiently characterized (Kazi et al. 2022, Fristot et al. 2024). In *L. lactis*, the nisin promoter (P*_nis_*) is extensively used and with some clear advantages over alternative inducible systems responsive to sugar, pH, metals and salt (Kok et al. 2017, de Castro et al. 2018). In beneficial streptococci, some inducible promoters have been adapted for expression studies (e.g. P*_lac_*, P*_xyl_*, and P*_tet_*) (Knoops et al. 2022a, b, Mignolet et al. 2018, 2019, Markakiou et al. 2023). Nonetheless, such controllable tools need to be transferred to other (pathogenic) streptococci, apart from *S. pneumoniae and Streptococcus pyogenes* (Lautenschlager et al. 2024), and to lactic acid bacteria in general.

The aim of this work is to expand and implement the molecular toolbox for streptococcal species and, to a broader scope, for lactic acid bacteria. Here, we make available FP-tagged HlpA^Sp^ constructs associated with four antibiotic resistances usable for streptococci (*hlpA^Sp^-fp*-Ab^R^ cassettes). The selected FPs (mTurquoise2, mNeonGreen, a variant of msfYFP, mScarlet-I and mKate2) allow simultaneous tripartite detection. To have the broadest application, the FP-fusion cassettes were cloned in *E. coli* on a high-copy number plasmid [pJet1.2/blunt, Thermo Scientific (Nawawi et al. 2022)] with a non-functional promoter sequence, because expression of *Streptococcus intermedius hlpA* is toxic in *E. coli* (Liu et al. 2008). The cassettes can be used for overlapping PCR engineering prior to chromosomal integration into naturally competent streptococcal strains or on shuttle vectors. As a proof of principle, *hlpA^sp^-fp-Ab^R^* cassettes were successfully transferred into a broad-range firmicute integrative shuttle vector. Furthermore, FPs were fused to proteins exhibiting specific subcellular localization (in addition to HlpA) and co-produced from endogenous, constitutive or inducible promoters. The molecular toolbox presented here will facilitate single cell analysis and gene expression studies in streptococci.

## Results and discussion

### Chromosomal insertion at the hlpA locus of *Streptococcus thermophilus*

**Figure 1:**
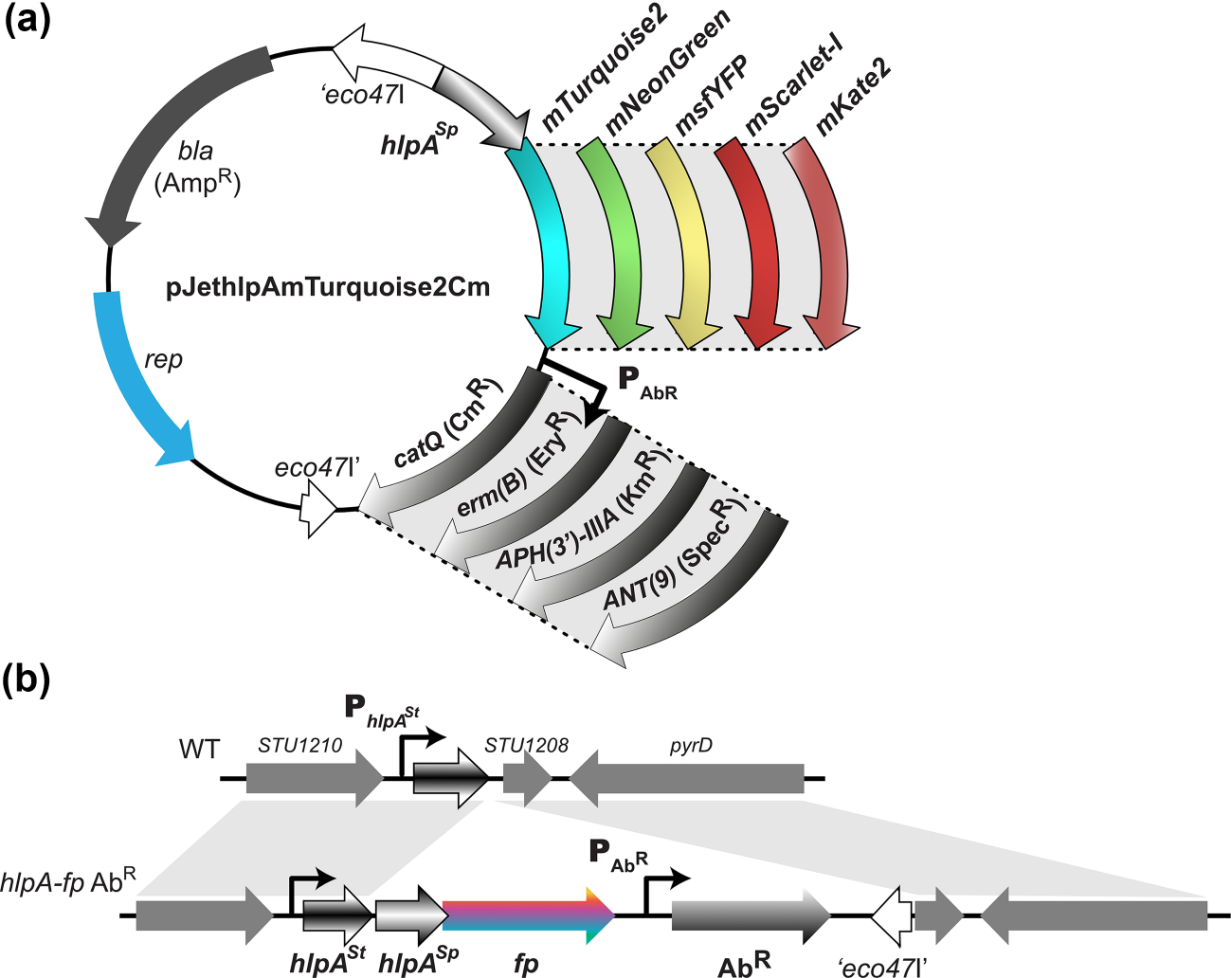
Engineering toolbox for gene insertion and fluorescent protein production in *S. thermophilus*. (a) General scheme of the pJethlpAmTurquoise2Cm and its derivatives. The plasmid includes the pBR322 origin (*rep*) for propagation in *E. coli*. The ampicillin resistance gene (*bla*) is shown . The *eco47*I gene is disrupted by the *hlpA^Sp^-fp* fusion, the antibiotic resistance genes (Ab^R^) cloned under their own promoter control and with transcriptional terminators. A set of 20 plasmids was created, each containing a different *hlpA^Sp^* chimeric gene linked to one out of five genes encoding fluorescent protein (mTurquoise2, mNeonGreen, msfYFP, mScarlet-I, and mKate2) and one out of four antibiotic resistance genes [*catQ, erm*(B), APH(3')-IIIa, ANT(9)]. (b) Homologous recombination at the *hlpA* locus of *S. thermophilus*. On the top panel, the recipient chromosomal locus of *S. thermophilus* LMG18311, *hlpA^St^* for *hlpA^Sp^-fp*-AbR cassette integration is shown. On the bottom panel, the final insertion product is depicted. After homologous recombination, the *hlpA^Sp^-fp*-AbR cassette is inserted downstream of the *hlpA^St^* gene, as highlighted by the homologous shaded regions. Promoters are marked with arrows symbols.

We selected the following five fluorescent proteins (FPs) for expression in streptococci: mTurquoise2, mNeonGreen, msfYFP, mScarlet-I, and mKate2. Genes encoding the FPs were previously codon-optimized for *S. pneumoniae* and shown to be well expressed in *S. pneumoniae* when fused at the C-terminus of HlpA (Kjos et al. 2015, Kjos 2019). The five *hlpA^Sp^-fp* fusions were amplified as PCR products and inserted into the pJet1.2/blunt positive selection vector. After electrotransformation in *E. coli*, we validated by sequencing that all vectors carried the correct *hlpA^Sp^-fp* insert downstream of the T7 RNA polymerase promoter. For selection and maintenance, resistance genes for chloramphenicol (*cat*Q; Cm^R^), erythromycin [*erm*(B); Ery^R^], kanamycin [APH(3')-IIIa; Kan^R^], and spectinomycin [ANT(9); Spec^R^] were cloned with their own promoters (functional in both *E. coli* and firmicutes) downstream of the *hlpA^Sp^-fp* fragments, ensuring the same transcriptional orientation (Fig. 1a). The constructed plasmids (available at Addgene #206810–206 829) are replicative only in *E. coli* (Supplementary Table S1) and can be used as templates for PCR amplification of *hlpA^Sp^-fp, fp-Ab^R^* or *hlpA^Sp^-fp*-Ab^R^ cassettes (primer sequences listed in the Supplementary Table S2).

The *S. thermophilus hlpA* chromosomal locus was previously characterized by Dixon-Fyle and Caro (1999) (Fig. 1b). As a first instance and proof of principle, insertion of the *hlpA^Sp^-fp-*Ab^R^ (homologous recombination arms of ∼1 kb) was performed following the strategy outlined by Kjos et al (2015). Since *hlpA* is essential in *S. pneumoniae* D39, *hlpA^Sp^-fp* was integrated downstream of the native *hlpA^St^* gene. Natural competence transformation combined with antibiotic selection using the *hlpA^Sp^-fp-*Ab^R^ cassettes, provided an efficient and time-saving method for strain modification (Fig. 1b, successful recombination events shown in gray). Transcription of *hlpA^Sp^-fp* was driven from the native *hlpA^St^* promoter, forming a single transcriptional unit with a duplication of the ribosomal binding site of *hlpA^St^*. Clones were verified by PCR and sequencing.

### Quantitative analysis of fluorescence detection in growing cultures of *S. thermophilus hlpA^Sp^-fp*

**Figure 2:**
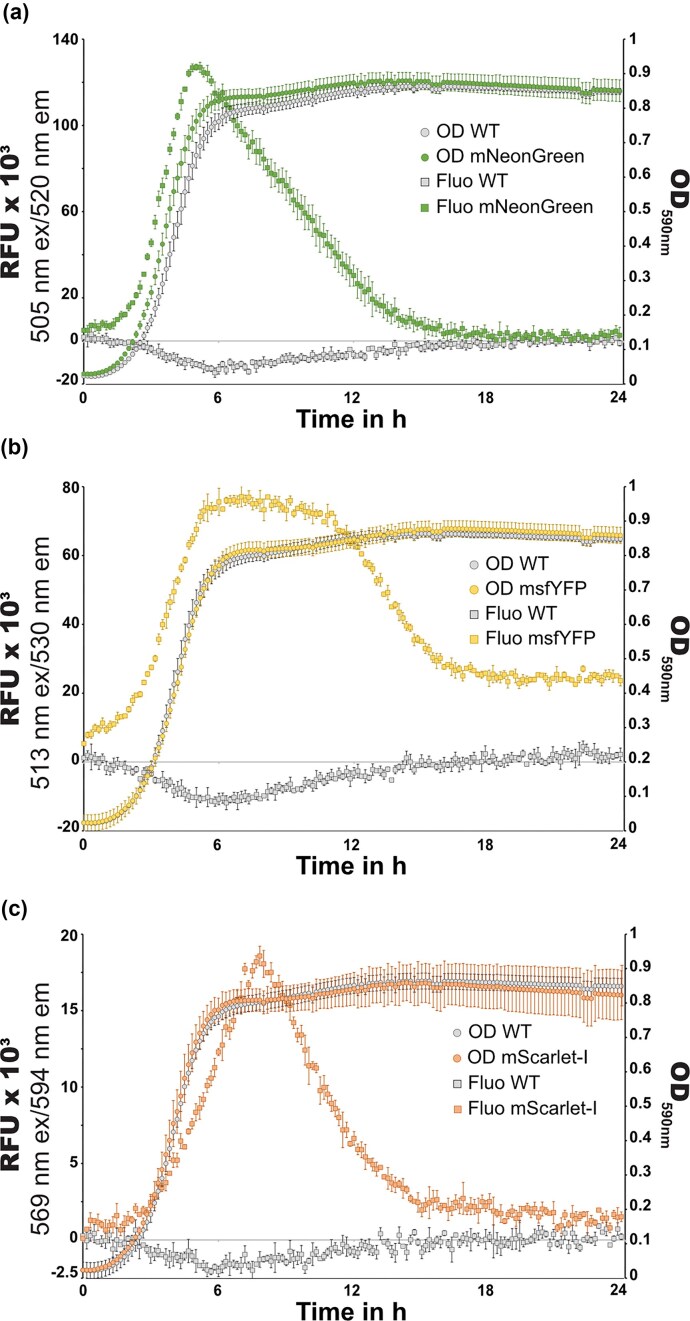
Fluorescence quantification of *S. thermophilus hlpA^Sp^-fp* during growth. (a-c) Fluorescence measurements of growing *S. thermophilus* LMG18311 (WT) and its derivative strains in M17L medium. Growth curves are assessed via culture turbidity at 590 nm (round symbols) and fluorescence measurements are expressed in relative fluorescence units (RFUs, after subtracted the M17L signal, square symbols). LMG18311 derivatives shown by colored symbols, while background fluorescence in the parental LMG18311 strain is plotted in grey. RFU measurements were taken at 505 nm ex/520 nm em (mNeonGreen) (a), 513 nm ex/530 nm em (msfYFP) (b), and 569 nm ex/594 nm em (mScarlet-I) (c). Due to significant background fluorescence for the M17L medium, the parental strain LMG18311 in m17L reduced the signal, resulting in negative values when the blank (M17L without cells) was subtracted (grey squares). Data points are the means from triplicate assays with error bars indicating the mean ± standard deviation.

To assess the fluorescence profiles of each FP fusion, cultures in early stationary phase were diluted in fresh M17L medium to a starting culture turbidity (OD at 590 nm) of 0.02. Growth and fluorescence signals were recorded every 10 min during 24 h in a microtiter plate assay (see Methods). Growth curves of the engineered strains were comparable to the parental LMG18311 strain, indicating that the integration of *hlpA^Sp^-fp* downstream of the *hlpA^St^* gene had no or minimal effect on growth (Fig. 2 and Supplementary Figure S1). No detectable fluorescence could be observed for mTurquoise2 and mKate2 in this medium and with our available microtiter plate setup (Supplementary Figure S1). However, high signals in the channels for mNeonGreen, msfYFP, and mScarlet-I fluorescence were detected during growth (Fig. 2a–c). Fluorescence levels continuously increased with cell density until the bacterial population reached the stationary phase. The signal peaked early in stationary phase for HlpA^Sp^-mNeonGreen (Fig. 2a) and HlpA^Sp^-msfYFP (Fig. 2b), and ∼2 h later for HlpA^Sp^-mScarlet-I (Fig. 2c), suggesting a longer maturation time of mScarlet-I in *S. thermophilus*, as previously reported for eucaryote and other bacterial expression systems (Bindels et al. 2017). Approximately 10 h after this peak value of fluorescence, the levels declined to the background level of the wild-type LMG18311 culture. There was some overlap between the HlpA^Sp^-msfYFP and HlpA^Sp^-mNeonGreen fusion protein fluorescence emissions (Supplementary Figure S2a-b). However, fluorescence measurements in relative fluorescent units (RFUs) for specific excitation and emission wavelengths of each protein showed a stronger signal in their selective channel. No cross-detection was observed between HlpA^Sp^-mScarlet-I and HlpA^Sp^-mNeonGreen or HlpA^Sp^-msfYFP suggesting that mScarlet-I and mNeonGreen/msfYFP can be simultaneously recorded in microtiter plate assays (Supplementary Figure S2a-c). Since the integration locus, promoter, and protein fusion design were identical for the three fluorescent proteins, the observed fluorescence curves likely reflect differences in the folding kinetics and stability of the HlpA^Sp^-FP fusions, which are directly influenced by the specific fluorescent proteins. These data are comparable to fluorescent proteins expressed in the (previously) bioengineered *S. pneumoniae* strains (Kjos and Veening 2014, Kurushima et al. 2020). First, expression of fluorescent fusions has no or minor impact on cell growth (Supplementary Figure S3a). Next, we observed no cross-detection between mScarlet-I and mNeonGreen/msfYFP fusions. Finally, the msfYFP associated signal remains at high level during an extended period of time compared to the mNeonGreen signal that decreases after 5 h and the mScarlet-I signal that displays a sharp peak (Supplementary Figure S3c and S3d). In contrast to *S. thermophilus*, expression of mTurquoise2 and mKate2 fusions produced a signal (even if very weak for mKate2) distinguishable from the WT non-fluorescent strain (Supplementary Figure S3b). This could be due to the different medium used for *S. pneumoniae* (C+Y) growth with less autofluorescence compared to the *S. thermophilus* growth medium (M17L medium).

### Effect of growth conditions on fluorescent signal intensity

Considering that *S. thermophilus* is described as an anaerobic, aerotolerant bacterium (Thibessard et al. 2004) that can acidify its environment during growth and often presented as a thermophilic bacterium (Zhang et al. 2011), we cultivated the LMG18311 *hlpA^Sp^*-*mNeonGreen, hlpA^Sp^-msfYFP* and *hlpA^Sp^-mScarlet-I* strains under oxygen-limiting conditions or we exposed them to different pH levels (pH 6, pH 7, and pH 8) and temperatures (25°C, 37°C, and 45°C). A fluorescent signal was detected for the three strains under all conditions tested and cultures increased in cell density until reaching the stationary phase (Supplementary Figure S4a-i). Similar profiles were observed for the three strains under oxygen-limiting conditions, with higher OD and a reduced fluorescent signal compared to aerobic conditions (Supplementary Figure S4a–c). These observations are consistent with previous data indicating that the fluorescent proteins require oxygen (O_2_) to mature the chromophore that produces light (Heim et al. 1994). Considering acido-basic conditions, the growth was globally hampered at pH 6 and pH 8 and a lower pH reduced the signal for all FPs (Supplementary Figure S4d-f). Moreover, HlpA^Sp^-mNeonGreen produced a sharper and higher peak at pH 7, while the msfYFP signal, detectable for more than 15 h after reaching the stationary phase, was 30% stronger at pH 8. Interestingly, HlpA^Sp^-msfYFP and HlpA^Sp^-mScarlet-I exhibited a bimodal signal at pH 8, suggesting a new wave of transcriptional activation at the *S. thermophilus hlpA* locus. Finally, the temperature experiments were highly consistent between FPs and shifting from the optimum temperature (37°C) decreased the growth rate, while higher temperatures exhibited overall higher fluorescent signals (Supplementary Figure S4g-i).

### Microscopy-based quantification of fluorescent signal in cultures of *S. thermophilus* producing HlpA^Sp^-FP

**Figure 3:**
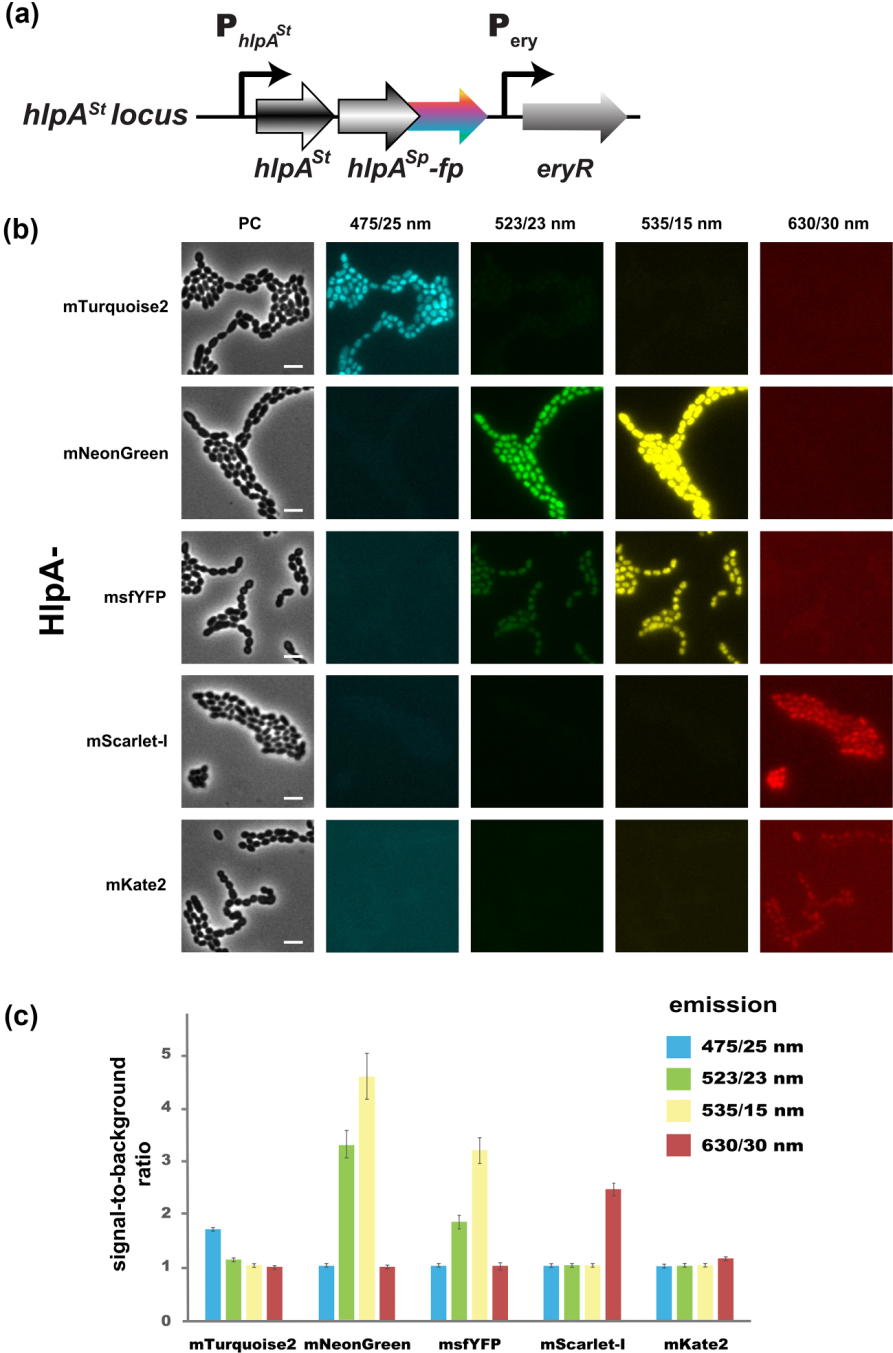
Single-cell visualization and quantification of FP signals in *S. thermophilus*. (a) Genetic structure of the *hlpA^Sp^-fp* integrated at the native *hlpA^St^* locus under the control of its own promoter. The Ery^R^ gene is used to select chromosomal integration of *hlpA^Sp^-fp* constructs. (b) Cell micrographs of *S. thermophilus hlpA^Sp^-fp* expressing strains for HlpA^Sp^-mTurquoise2, HlpA^Sp^-mNeonGreen, HlpA^Sp^-msfYFP, HlpA^Sp^-mScarlet-I or HlpA^Sp^-mKate2 (as indicated on the left) in the respective fluorescence channels, or in phase contrast (PC). Images are scaled to same brightness and contrast. Scale bars represent 2 µm. (c) Signal-to-noise ratio for the five FPs of panel (b) in *S. thermophilus* in their cognate emission channels: 480/40 nm (blue), 525/50 nm (green), 535/50 nm (yellow), and 630/60 nm (red). Cells were segmented with Fiji to quantify the mean fluorescence pixel intensity per cell, which is divided by the mean pixel intensity of image background (excluding cells). Bars represent the means ± standard error from three images.

**Figure 4:**
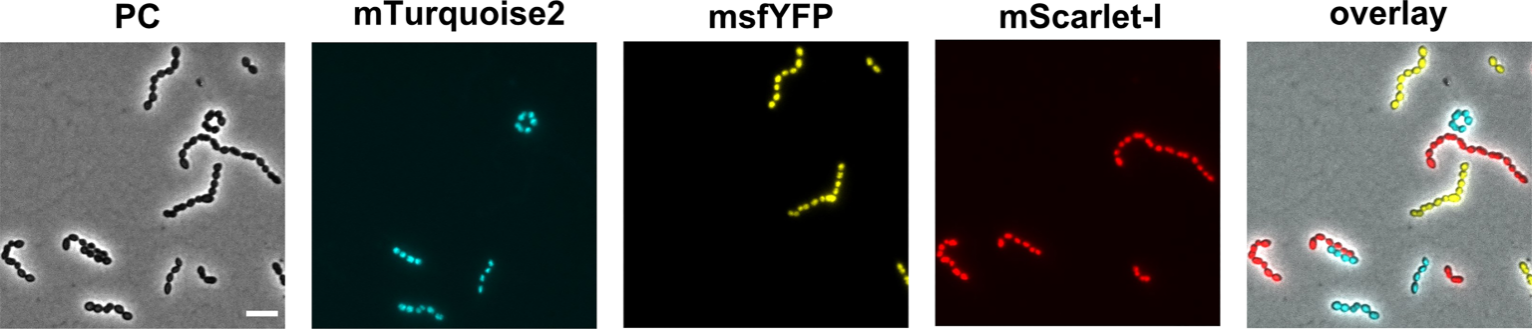
Fluorescence-based discrimination of mixed *S. thermophilus* populations producing HlpA^Sp^-FP fusions. Cell micrographs show an equal proportions mixture of three *S. thermophilus* LMG18311 populations expressing HlpA^Sp^-mTurquoise2, HlpA^Sp^-msfYFP or HlpA^Sp^-mScarlet-I, imaged in phase contrast (PC), or by the specific fluorescence settings for each of the FPs (485/40 nm, 535/50 nm, and 630/60 nm, respectively), along with a color overlay image. Scale bar represents 2 µm.

Epifluorescence microscopy of *S. thermophilus* cells producing various HlpA^Sp^-FP fusions allowed us to define the signal-to-noise ratio (mean intensity of the cellular area divided by the mean intensity of the non-cell background area in a specific channel) for each FP with its respective filter. Light source intensity and exposure time were adjusted to minimize photobleaching for each individual fusion. As shown in Fig. 3a and b, the mTurquoise2, mNeonGreen, msfYFP, and mScarlet-I HlpA fusions produced a high fluorescence signal that was restricted to the bacterial nucleoid, as expected for a non-specific nucleoid binding protein, while the signal associated with the mKate2 fusion was dimmer (lower signal-to-background ratio compared to mScarlet-I) (Fig. 3c). Moreover, recording the fluorescence values and evaluating the signal-to-noise ratio of each fusion for each set of filters showed that the HlpA^Sp^-mTurquoise2 fusion is fully compatible with every other fusion (no overlap between channels) in *S. thermophilus* (Fig. 3b). By contrast, two pairs of FPs, HlpA^Sp−^mNeonGreen/HlpA^Sp^-msfYFP and HlpA^Sp^-mScarlet-I/HlpA^Sp^-mKate2, could not be differentiated specifically and were incompatible in our microscope setup. However, their signals did not bleed through other channels, meaning they can be imaged with all other FPs tested here. To conclude, the nucleoid-associated signal of HlpA^Sp^-FP fusions (rather than a diffuse cytoplasmic fluorescence for FPs alone) enables clear identification of cells even at low signal intensities. In *S. thermophilus*, HlpA^Sp^-mTurquoise2, HlpA^Sp^-msfYFP and HlpA^Sp^-mScarlet-I emit distinct and compatible fluorescent signals, when detected with their respective filters, allowing accurate identification and enumeration of individual cells in a mixed population (Fig. 4).

### Constitutive *hlpA^sp^-fp* expression from ectopic locus in *Streptococcus salivarius*

**Figure 5:**
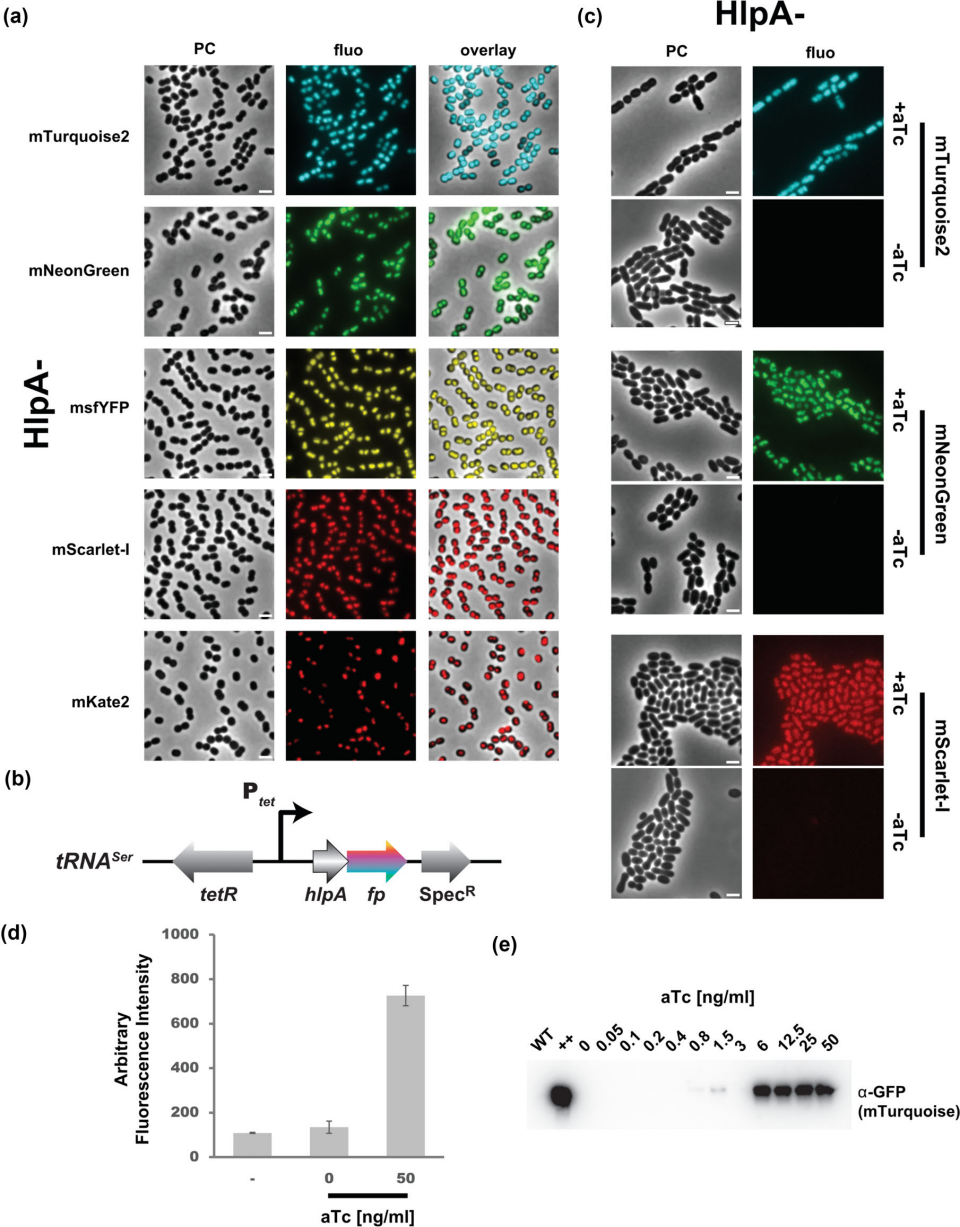
Constitutive and inducible nucleoid-FP labeling in *S. salivarius*. (a) Micrographs of *S. salivarius* strains expressing the HlpA^Sp^ nucleoid-associated factor FP fusion for each of the fluorescent protein variants, as indicated on the left, imaged by phase contrast (PC), or fluorescence recorded in the cognate channel, and an overlay. The scale bars represent 2 µm. (b) Scheme of the genetic structure of the P*_tet_*-inducible *hlpA^Sp^-fp* integrated at the *tRNA^Ser^* locus. The *tetR* repressor gene, with its own promoter (not depicted here), points in the opposite orientation compared to the P*_tet_*. The Spec^R^ gene is used to select chromosomal integration of *hlpA^Sp^-fp* constructs. (c) Single-cell imaging of aTc-inducible tagged HlpA^Sp^-FP with mTurquoise2, mNeonGreen or mScarlet-I in *S. salivarius* cells, incubated with (+) or without (−) 50 ng.ml^−1^ aTc for 2 h. All images are scaled to the same brightness. The scale bars equal 2 µm. (d) Mean single cell fluorescence of the aTc-induced (50 ng.ml^−1^) or uninduced (0) *hlpA^Sp^-mTurquoise2* strain of *S. salivarius*, in comparison to the wild-type non-labeled HSISS4 strain as a negative control (−). Bars show the mean ± standard error from cells segmented on three separate images. (e) Anti-GFP antibody stained immunoblot showing the HlpA-mTurquoise2 levels upon growth in M17G as a function of aTc concentration (from 0.05 to 50 ng.ml^−1^). WT, a non-labeled control strain; (++), a constitutive overexpression mutant (expression from P_32_).

To validate that the HlpA^Sp^-FP fusions are exploitable in other species, we adapted the system to *Streptococcus salivarius*, a human commensal that is highly prevalent in the digestive tract. *S. salivarius* is genetically amenable due to its natural competence but fluorescent tools to perform single-cell analyses and monitor population dynamics are missing. To probe the versatility of our genetic toolbox, we decided to use an alternative strategy to clone the fluorescent reporters (constitutive or inducible promoters, endogenous tagged proteins, different antibiotic cassettes, and ectopic insertion loci). We inserted the *hlpA^Sp^-fp* constructs at a non-essential ectopic locus in *S. salivarius* next to *tRNA^Ser^* and expressed them under the control of the constitutive P_32_ promoter (Mignolet et al. 2018) (Supplementary Figure S5a). The continuous production of these fusions has no major impact on cell growth (Supplementary Figure S5b). The mTurquoise2, mNeonGreen, msfYFP, mScarlet-I, and mKate2 fusions produced a strong homogeneous signal localized to the nucleoid (Fig. 5a and Supplementary Figure S5c), well distinguishable above background (Supplementary Figure S5d). Moreover, most pairs are compatible for multi-labeling, and the trio mTurquoise2/mNeonGreen/mScarlet-I enables simultaneous imaging with high signal-to-noise ratio and minimal crosstalk in *S. salivarius* on a standard widefield epifluorescence microscope.

### Ectopic chromosomal expression under inducible promoter control in *S. salivarius*

Misexpression at an inappropriate time of the cell cycle or cell growth might turn out to have toxic side effects. Therefore, we evaluated several inducible promoters for FP expression in *S. salivarius* that could be operated simultaneously. Two xylose-responsive promoters with distinct strength and leakiness have previously been reported for *S. salivarius* (Mignolet et al. 2018). As further inducible promoter we adapted the tetracycline-responsive promoter PT5-3 (hereafter referred to as P*_tet_*) that was optimized for tightness and strength in *S. pneumoniae* (Sorg et al. 2020). Transformation products were assembled by overlapping PCRs to replace the P_32_ promoter with P*_tet_* upstream of the mTurquoise2, mNeonGreen or mScarlet-I fusion genes at the *tRNA^Ser^* locus (Fig. 5b). Induction for 4h30 with a saturating concentration of anhydrotetracycline (aTc; 50 ng.ml^−1^) to derepress P*_tet_* caused a strong, homogeneous signal under the microscope for each of the three constructs (Fig. 5c). In contrast, cells grown in the absence of aTc emitted fluorescence close to the non-cell background. Quantification of P*_tet_*-*hlpA^Sp^-*mNeonGreen fluorescence under aTc-induction showed a 5.4-fold increase compared to uninduced cells (Fig. 5d). Western blotting confirmed induction of HlpA^Sp^-mTurquoise2 in *S. salivarius* as a function of aTc concentrations (Fig. 5e), saturating above an aTc concentration of 6 ng.ml^−1^ and decreasing to background in a non-mTurquoise2 WT strain at 0.4 ng.ml^−1^. This range of aTc concentrations is similar to induction curves previously reported for *S. pneumoniae* (Sorg et al. 2020). However, in comparison to the same protein fusion under constitutive P_32_ expression (++; Fig. 5e), the level of HlpA^Sp^-mTurquoise2 from P*_tet_* at saturating concentrations of aTc was markedly lower. In conclusion, the aTc-inducible promoter in *S. salivarius* allows a titratable, robust, and mild-to-strong protein expression. Initially implemented in *S. pneumoniae*, it can be transferred to other phylogenetically distant streptococci such as *S. salivarius*, and possibly outside of the *Streptococcus* genus.

### Fluorescently tagged proteins to image and track subcellular localization in *S. salivarius*

**Figure 6:**
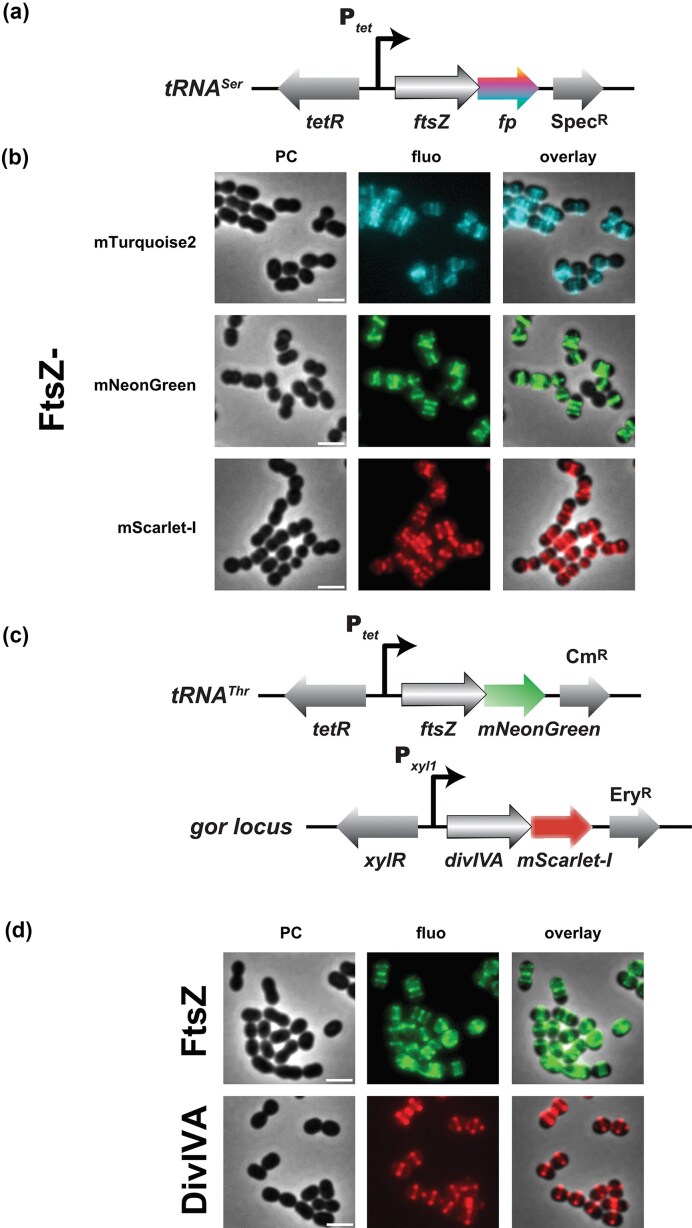
Subcellular localization of FtsZ and DivIVA in *S. salivarius*. (a) Genetic structure of the P*_tet_*-inducible *ftsZ-fp* integrated at the *tRNA^Ser^* locus. (b) Micrographs of single cells of aTc-induced FtsZ-FP (mTurquoise2, mNeonGreen or mScarlet-I; 50 ng ml^−1^ aTc for 2 h before imaging). Label indications and scale bar as before (2 µm). (c) Genetic structures of the P*_tet_*-inducible *ftsZ-mNeonGreen* integrated at the *tRNA^Thr^* locus and the P*_xyl1_*-inducible *divIVA-mScarlet-I* integrated at the *gor* locus in *S. salivarius*. (d) Micrographs of single cell aTc-derepressed FtsZ-mNeonGreen and xylose-induced DivIVA-mScarlet-I fusions, after incubation with the inducers for 2 h before imaging. The scale bars equal 2 µm.

We wondered if we could exploit mTurquoise2, mNeonGreen, and/or mScarlet-I fusions to follow protein dynamics at the single-cell level in *S. salivarius*. A highly dynamic and nearly universal landmark for the future site of division in bacteria is the so-called Z-ring formed by the tubulin-like protein FtsZ (Cameron and Margolin 2024). To image the division site in *S. salivarius*, we engineered strains to produce FP-tagged fusions of *S. salivarius* FtsZ. Considering that constitutive overexpression of *ftsZ* is likely to alter the Z-ring polymerization state and might be deleterious to cell shape (Ward and Lutkenhaus 1985), we used the *tRNA^Ser^* platform to ectopically integrate *ftsZ-fp* under the inducible P*_tet_* control as a second copy of *ftsZ* (Fig. 6a). As shown in Fig. 6b, all FtsZ-FP fusions formed well defined rings at the midcell, in line with what was observed for *S. pneumoniae* (Fleurie et al. 2014a, Morlot et al. 2003, Gallay et al. 2021). As cells entered the late phase of cytokinesis, the FtsZ fluorescent signal faded away from the equatorial plane and new Z-rings appeared at the quarter positions representing the future division sites (Movie S1). These experiments validate that *S. salivarius* has a division mode similar to *S. pneumoniae* in which cells elongate and divide in a single plane, in contrast to true cocci like *Staphylococcus aureus* (Pinho and Foster 2024).

### Monitoring protein choreography in real-time with multi-labeling

To monitor the interplay between two or more proteins, multicolor labeling can be used to assess protein localization simultaneously in the same cell. We thus wanted to create a strain with multiple simultaneous mTurquoise2, mNeonGreen, and mScarlet-I fusions to three different proteins in single living *S. salivarius* cells. Besides FtsZ and HlpA, we selected DivIVA, as it partially co-localizes with FtsZ in *S. pneumoniae* (Fadda et al. 2007). DivIVA is a membrane-tethered hub in Gram-positive bacteria that accumulates at the septum, as well as at both cell poles due to its high affinity for negatively curved membranes (Lenarcic et al. 2009, Vollmer et al. 2019).

First, we individually evaluated DivIVA localization from the different FP fusions, because previous results in *S. pyogenes* or *Streptomyces coelicolor* indicated that DivIVA incorrectly localizes upon overexpression provoking abnormal cell morphologies (Hempel et al. 2008, Raz et al. 2012). We first engineered constitutively expressed (P_32_) fusions integrated into the chromosome at the ectopic *tRNA^Ser^* locus (Supplementary Figure S6a). Akin to its profile in *S. pneumoniae* (Fleurie et al. 2014b, Trouve et al. 2024), *S. salivarius* DivIVA localized to the midcell, and in a few cases to the cell poles (Supplementary Figure S6b). When integrated at the *tRNA^Ser^* locus and expressed under the control of two xylose-inducible promoters (P*_xyl1_* and P*_xyl2_*) (Mignolet et al. 2018) (Supplementary Figure S6c), we observed fluorescent DivIVA at the equatorial planes and at cell poles in *S. salivarius* cells (Supplementary Figure S6d). Expression from P*_xyl1_* resulted in higher fluorescence compared to P*_xyl2_* but morphological defects were not noticeable.

**Figure 7:**
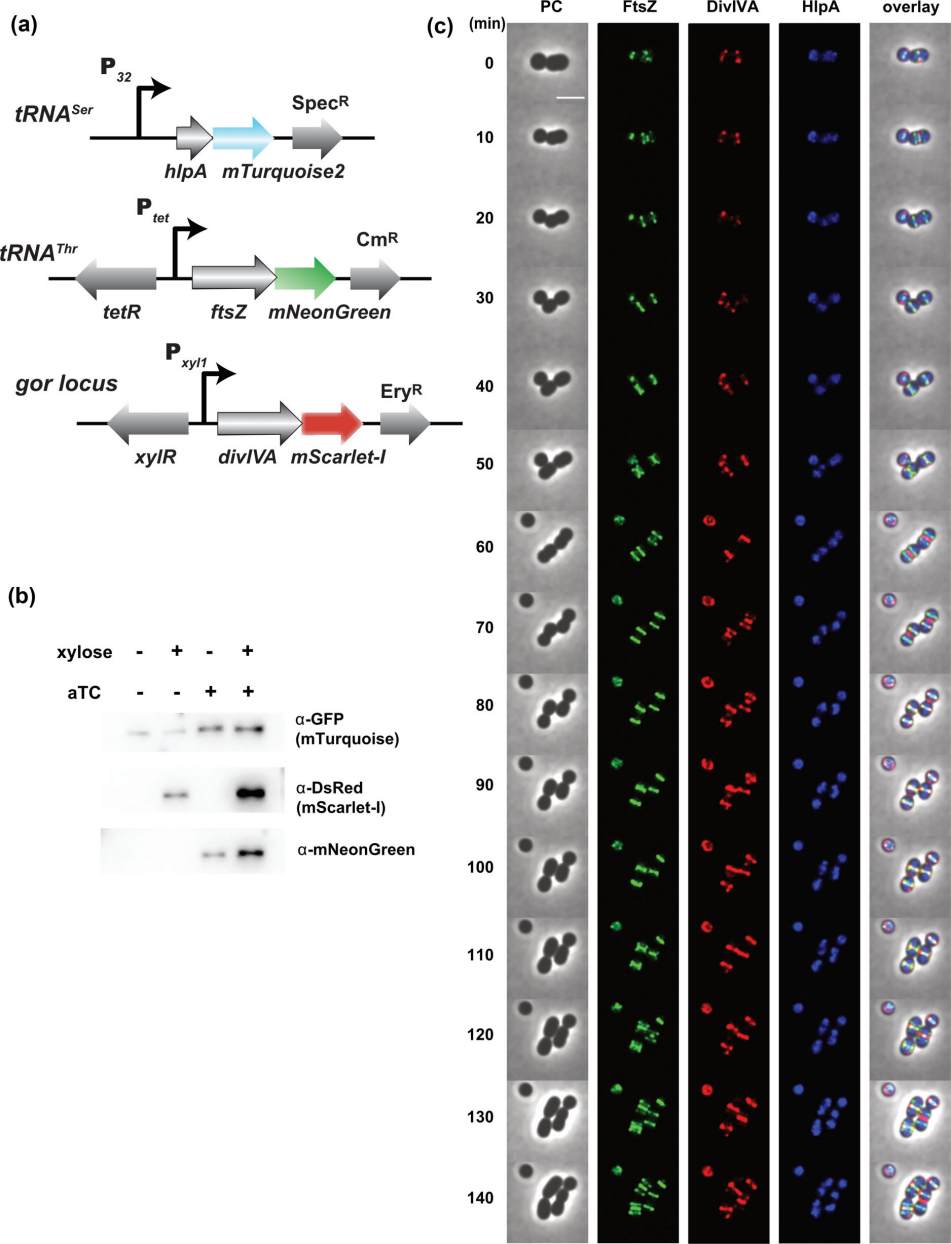
Dynamic localization of FtsZ-, DivIVA- and HlpA-fusions in *S. salivarius* cells by coexpressed fluorescent proteins. (a) Genetic constructions of the multi-labeled *S. salivarius* strain. *hlpA-mTurquoise2* was integrated at the *tRNA^Ser^* locus under the control of the constitutive P*_32_* promoter, while the P*_tet_*-inducible *ftsZ-mNeonGreen* and the P*_xyl1_*-inducible *divIVA-mScarlet-I* were integrated at the *tRNA^Thr^* and *gor* locus, respectively. (b) Immunoblot showing the HlpA-mTurquoise2, FtsZ-mNeonGreen, and DivIVA-mScarlet-I production after growth in M17G with (+) or without (−) inducers (aTc and/or xylose). The mTurquoise2, mScarlet-I and mNeonGreen fusions were detected with an anti-GFP, anti-DsRed and anti-mNeonGreen primary antibodies, respectively. (c) Time-lapse images at 10 min intervals of the multi-labeled strain HlpA^Sp^-mTurquoise2/FtsZ-mNeonGreen/DivIVA-mScarlet-I, after incubation with aTc and xylose for 2 h before imaging. The inducers were supplemented in the agarose pad used to support the cells for imaging. Fluorescent images were deconvoluted with the Huygens software and assembled with Fiji. The scale bar equals 2 µm.

Next, we selected a combination of FtsZ-mNeonGreen, HlpA^Sp^-mTurquoise2 and DivIVA-mScarlet-I fusions in a unique triple label strain. To concomitantly observe the profiles of three distinct fusion proteins, we kept the P*_32_*-*hlpA^Sp^-mTurquoise2* construct at the *tRNA^Ser^* locus and transferred both the P*_tet_-ftsZ-mNeonGreen* and P*_xyl1_-divIVA-mScarlet-I* constructs to other chromosomal loci with different antibiotic markers. As integration platforms, we used two previously reported permissive loci *tRNA^Thr^* (Mignolet et al. 2018) and *gor* (Knoops et al. 2022a) (Fig. 6c). In a validation experiment, we observed each fluorescent fusion separately (Fig. 6d). Induction of each fusion produced a similar localization profile compared to the fusions integrated at the *tRNA^Ser^* locus (Fig. 6b and S6d). Therefore, we engineered a chassis that encodes the FtsZ, DivIVA and HlpA fusions (strain VL5940: P_32_-*hlpA^Sp^-mTurquoise2*, P*_tet_-ftsZ-mNeonGreen*, P*_xyl1_-divIVA-mScarlet-I*) and performed a time-lapse experiment to monitor protein localization over time intracellularly (Fig. 7a). Western blotting showed that the fusion proteins were correctly produced, either constitutively (HlpA^Sp^-mTurquoise2), or specifically produced upon induction with aTc and xylose for FtsZ-mNeonGreen and DivIVA-mScarlet-I, respectively (Fig. 7b and Supplementary Figure S7). As expected, HlpA formed nucleoid-like condensed clusters excluded from the constriction site at late stages of division, while FtsZ and DivIVA moved from the midcell to the next area of septal formation (Fig. 7c; Supplementary Movie S1). However, the timing of relocation differed between FtsZ and DivIVA, similar to the temporal hierarchy observed in *S. pneumoniae* (Beilharz et al. 2012). Indeed, DivIVA colonized the midcell with a small delay and dwelled at this location for a longer time compared to FtsZ. All together, these data show that co-visualization of multiple proteins with different FPs at single cell level can be achieved in *S. salivarius*. The specific protein labeling here allowed us to refine the sequential recruitment steps of its cell division proteins in spatial context.

### Integrative plasmid tools for non-naturally transformable streptococci

**Figure 8:**
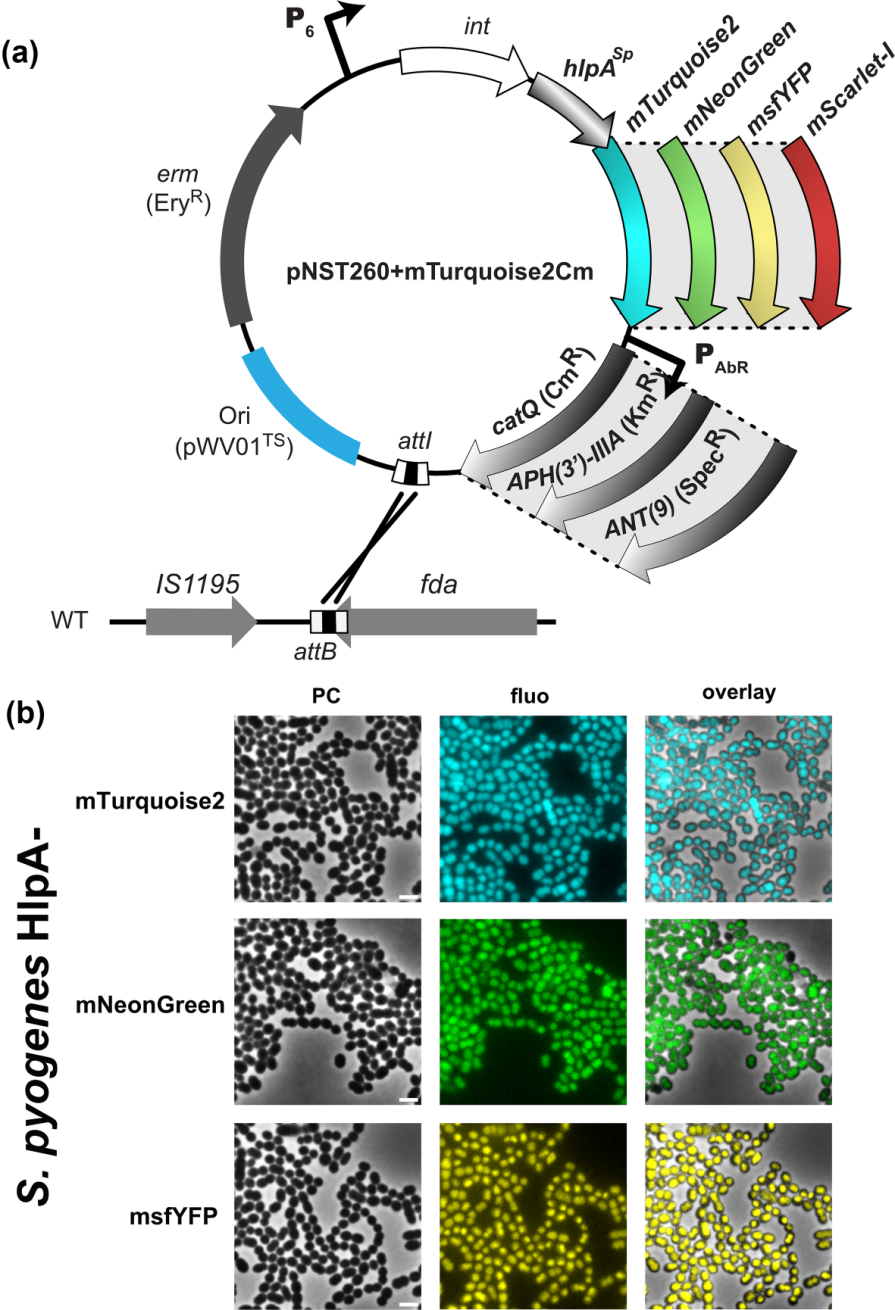
Constitutive nucleoid-FP labeling in non-naturally transformable streptococci. (a) General scheme of the pNST260+mTurquoise2Cm and its derivatives. The plasmid includes the pWV01 thermosensitive origin of replication (in blue). The erythromycin resistance gene (*erm*) is shown in grey. The *hlpA^Sp^-fp* fusions and associated antibiotic resistance genes are cloned in between the integrase gene (*int*) under the control of the constitutive P_6_ promoter and the integration site (*attI*). A set of 12 plasmids was created, each containing a different *hlpA^Sp^* chimeric gene linked to one out of four genes encoding fluorescent protein (mTurquoise2, mNeonGreen, msfYFP, and mScarlet-I) and one out of three antibiotic resistance genes [*catQ*, APH(3')-IIIa, ANT(9)]. Directed recombination (symbolized with X) occurs at the integration site (*attB*) in the *fdA* locus of *S. salivarius* chromosomes. Promoter is marked with an arrow symbol. (b) Micrographs of *S. pyogenes* strains expressing the HlpA^Sp^ nucleoid-associated factor FP fusion for each of the fluorescent protein variants, as indicated on the left, imaged by phase contrast (PC), or fluorescence recorded in the cognate channel, and an overlay. The scale bars represent 2 µm.

Linear DNA fragments cannot be easily integrated into the chromosome of species/strains unable to develop competence for natural transformation. Therefore, we designed fluorescence-based integrative plasmids that could be introduced by electroporation and maintained in the population via single chromosomal recombination. We decided to exclude replicative plasmids from our strategy considering that they can cause toxicity due to high protein production or cell heterogeneity due to variability in their copy number. We cloned the *hlpA^Sp^-fp-*Ab^R^ cassettes (where Ab^R^ is Cm^R^, Kan^R^, or Spec^R^) from our pJet constructs into the Ery^R^ pNST260+ backbone (Bellanger et al. 2009) to generate 12 new vectors (available at Addgene #253369–253 380) (Fig. 8a). The pNST260+ bears the integration site (*attI*) from ICE*St*1 and encodes the cognate integrase gene (*int*) to allow the irreversible recombination at the 3’ end of the *fda* gene (fructose-1,6-biphosphate aldolase) in a broad range of lactic acid bacteria and firmicutes (Burrus et al. 2002). Downstream of the *int* gene, an RBS sequence is cloned upstream of the *hlpA^Sp^-fp* fragment to form an operon under the control of the constitutive P_6_ promoter of *Lactobacillus acidophilus* (Djordjevic and Klaenhammer 1996), allowing the constitutive production of HlpA^sp^-FP fusions. Integration duplicates the 3’ end of *fba* and therefore does not cause deleterious effects on host physiology. Moreover, the thermosensitive origin of replication allows for selection of stably integrated plasmids in the chromosome when cells are shifted from 30°C to 37°C (Fig. 8a). We first validated our constructs in *S. salivarius* (Supplementary Figure S8a). Like our previous data, the fluorescent fusions produce a compact signal associated with the nucleoid in all visualized cells. Although cells appear to be slightly autofluorescent in the mTurquoise2 channel, the mNeonGreen/mScarlet-I and msfYFP/mScarlet-I pairs can be used for multi-labeling images. We next transformed the pNST vectors with the non-naturally transformable human pathogen *S. pyogenes*. We electroporated the integrative plasmids and verified chromosomal insertion via PCR. We imaged similar fluorescence profiles with *S. pyogenes* for mTurquoise2, mNeonGreen, and msfYFP fusions (Fig. 8b & Supplementary Figure S8b), strongly indicating that our ICE-based strategy can be used broadly with species containing an *attB* site in their genome.

## Conclusion

This study was based on the fluorescent labeling strategy proposed by Kjos et al. (2015), which involved operon insertion of *hlpA^Sp^-fp* constructs downstream of the endogenous *hlpA^St^* gene. This approach, optimized in *S. pneumoniae*, allowed constitutive fluorescent labeling without affecting cell morphology or growth, making HlpA^Sp^-FP a reliable nucleoid, cell and population marker. Using a detailed construction protocol (Kjos 2019), which employs PCR amplification of five fragments, we streamlined the integration process into *S. thermophilus* and *S. salivarius* by employing a simplified three-fragment overlapping PCR strategy. Fluorescence and antibiotic resistance genetic modules can be amplified from the 20 plasmids deposited at Addgene (Supplementary Table S1) and from the bioengineered strains (Supplementary Table S4) to produce fluorescently-labeled bacterial strains of interest. Finally, 12 ready-to-use labeling shuttle vectors carrying HlpA^Sp^-FP fusions for a wide range of firmicutes were generated, providing an important new platform for genetic manipulation in streptococci.

We validated the use of HlpA^Sp^ fused to various fluorescent proteins (mTurquoise2, mNeonGreen, msfYFP, mScarlet-I, and mKate2) in three streptococcal species and demonstrated their utility as nucleoid-localized markers across the different growth phases without detrimental effects (in the tested conditions) on cell morphology or fitness in streptococci. We also showed how regulatable promoters such as the anhydrotetracycline-derepressed (P*_tet_*) and the xylose-inducible (P*_xyl1_* and P*_xyl2_*) promoters enabled precise temporal control of visualized protein production, minimizing the metabolic burden associated with constitutive overproduction (see a summary in the Supplementary Table S6). Indeed, the transfer of these fluorescent and expression tools to other lactic acid bacteria will require careful individual validations. As shown in previous research, anoxic environment or acidity are likely to reduce or totally prevent folding or maturation of fluorescent fusions (Baird et al. 2000, Perez-Arellano and Perez-Martinez 2003, Landete et al. 2015), while some rich media or cytosolic constituents might increase the background noise and conceal the actual fluorescent signal (Stojanov et al. 2021). Moreover, overexpression of (DNA-binding) proteins could impose a metabolic/physiologic hindrance affecting bacterial growth, especially in the case of multi-labeling where anormal dosage/function (due to extra copies or fusions with tags) of endogenous proteins might also collide with essential cellular processes (Abdelaal and Yazdani 2021). Finally, the usage of plasmid-borne tags (instead of chromosomally encoded fusions) increases the variability of the signal due to fluctuation in the number of fluorescence gene copies (Jahn et al. 2016). Despite all these limitations, this study serves as a starting point to easily test the amenability of these tools in other bacterial species of scientific, clinical or commercial interest.

Finally, we further demonstrated the potential of multi-labeling approaches, with distinct populations of HlpA-FP-labeled cells being distinguishable and allowing for simultaneous monitoring of up to three proteins or cell populations. The utilization of spectrally distinct and photostable FPs provides a significant advantage for studying streptococci, whose small, chain-forming cells present unique challenges for live-cell imaging. Multicolor images enable tracking of individual cells and across chains with detailed analysis of division machinery, cell wall synthesis, and subcellular organization. It also facilitates observation of transient events such as competence development or quorum sensing, revealing heterogeneity that single-color approaches cannot capture. Furthermore, the improved brightness and spectral separation of the newly described FPs reduce background interference and autofluorescence, enhancing the resolution of dynamic processes such as biofilm formation and virulence factor localization. Together, these features make multi-labeling with optimized FPs a powerful tool for dissecting the complex spatial and temporal biology of streptococci.

The arsenal developed here provides a robust and versatile molecular toolbox, facilitating detailed studies of cellular dynamics and population behaviors in streptococci and probably beyond. These findings pave the way for broader applications in microbiology, including studies involving multiple labeled populations and dynamic protein interactions.

## Experimental procedures

### Bacterial strains and growth conditions

Bacterial strains used in this study are listed in the Supplementary Table S4. *Escherichia coli* was grown in LB medium with shaking at 37°C (Malke et al. 1990). *Streptococcus thermophilus* LMG18311 and its derivatives were grown at 37°C in M17L broth (M17: Difco Laboratories Inc., MI with 1% lactose [w/v]) or in half milk (Dorrazehi et al. 2020) without shaking. *Streptococcus salivarius* HSISS4 and its derivatives were grown at 37°C in M17G broth or CDMG (M17 and CDM broth containing 1% glucose [w/v], respectively) without shaking (Mignolet et al. 2018). *Streptococcus pneumoniae* D39V and derivatives were grown at 37°C in C+Y broth without shaking (Burnier et al. 2026). *Streptococcus pyogenes* NV28 and derivatives were grown at 37°C in Todd Hewitt Broth (Biocore Diagnostics) without shaking. When required, ampicillin (100 µg.ml^−1^ for *E. coli*), chloramphenicol (100 µg.ml^−1^ for *E. coli*, 4 µg.ml^−1^ for *S. thermophilus*, 5 µg.ml^−1^ for *S. salivarius*, and 2 µg.ml^−1^ for *S. pyogenes*), erythromycin (150 µg.ml^−1^ for *E. coli*, 5 µg.ml^−1^ for *S. thermophilus*, and 10 µg.ml^−1^ for *S. salivarius*), kanamycin (50 µg.ml^−1^ for *E. coli*, 500 µg.ml^−1^ for *S. thermophilus*, and 400 µg.ml^−1^ for *S. pyogenes*), spectinomycin (50 µg.ml^−1^ for *E. coli*, 500 µg.ml^−1^ for *S. thermophilus*, 200 µg.ml^−1^ for *S. salivarius* and 200 µg.ml^−1^ for *S. pyogenes*), anhydrotetracycline (aTc; 50 ng.ml^−1^ for *S. salivarius*) or xylose (1% for *S. salivarius*) were added to the media. Synthetic peptides (95% purity) including XIP (comX-inducing peptide for *S. thermophilus*) and sComS (for *S. salivarius*) were obtained from Peptide2.0 Inc. (Chantilly, VA, USA) and dissolved in DMSO. Plates inoculated with *S. thermophilus* or *S. salivarius* cells were incubated anaerobically (BBL GasPak systems, Becton Dickinson, NJ) at 37°C.

*Streptococcus thermophilus* and *S. salivarius* transformation protocols were adapted from Dorrazehi et al. (2020) and Mignolet et al. (2018), respectively. Briefly, overnight cultures of *S. thermophilus* were diluted 1:20 and incubated for 75 min at 37°C. Then, 1 µM of XIP and 5 µl of DNA cassettes (from overlapping PCRs) were added, followed by incubation for 3 h at 37°C before plating on selective M17L agar. Transformants were transferred to liquid selective M17L and after growth, re-isolated on selective solid medium. For *S. salivarius*, cultures were initiated in CDMG and incubated for 3.5 h at 37°C. Then, 1 µM sComS and DNA (from overlapping PCRs), were added, and cells were allowed to recover for 3 h at 37°C before plating on M17G agar supplemented with antibiotics when required.

*S. pyogenes* electroporation was performed as previously described (Bjanes et al. 2024).

### Construction of *S. pneumoniae* hlpA-fp

To generate *hlpA-mTurquoise2* and *hlpA-mNeonGreen* constructs in *S. pneumoniae*, the *hlpA-mTurquoise2* and *hlpA-mNeonGreen* genes were integrated downstream of the native *hlpA* gene as a second copy of *hlpA*, as previously described for *hlpA*-*mKate2* (Kjos and Veening 2014) and *hlpA*-*mScarlet-I* (Kurushima et al. 2020). The up and down homology regions were amplified by PCR using genomic DNA from VL1459 (*hlpA:: hlpA*_*hlpA*-*mKate2*-Cm^R^) (Kjos and Veening 2014) as a template, and using the primer pairs hlpA-up-F/hlpA-up-R and cam-hlpA-down-F/cam-hlpA-down-R, respectively. The *mTurquoise2* and *mNeonGreen* genes were PCR-amplified with the primer pairs mTurquoise2-F/mTurquoise2-R and mNeon-F/mNeon-R, respectively. We used codon-optimized synthetic *mTurquoise2* and *mNeonGreen* genes as templates (Keller et al. 2019). The resulting PCR fragments (see Supplementary Tables S5) were fused by overlapping PCR to generate the final constructs and transformed into *S. pneumoniae* D39V, as previously described (Burnier et al. 2026).

### Construction of pJet1.2 plasmid derivatives

Plasmids and oligonucleotides used in this study are listed in Supplementary Tables S1 and S2, respectively. Fluorescent tag properties used in this study are listed in Supplementary Table S3. Construction of *hlpA^Sp^-fp* plasmids was performed using the CloneJet PCR Cloning kit (Thermo Fisher Scientific, France) to ligate *hlpA^Sp^-fp* PCR fragments into the pJet1.2/blunt positive-selection cloning vector. Genomic DNA templates to amplify *hlpA^Sp^-fp* fragments were extracted from VL1778 (mTurquoise2), VL877 (mNeonGreen), VL1634 (msfYFP), VL1780 (mScarlet-I), or VL1459 (mKate2) with the primers hlpA_LB_F/Cm-int-R. The resulting recombinant plasmids contained *hlpA^Sp^-fp* inserts downstream of the T7 RNA promoter and disrupted the lethal restriction gene *eco*47I. *E. coli* strain DH5α (Invitrogen) was used as the host for plasmid amplification during cloning experiments. Electrotransformations were performed as described previously (Dower et al. 1988). All PCR reactions were performed with the high-fidelity DNA polymerase, Phusion (ThermoScientific) according to the manufacturer’s guidelines. A fragment containing APH(3’)-IIIa (Km^R^) with its promoter was amplified from the genomic DNA of strain LMG18311kan (Carraro et al. 2016) with the primer pair EcoRIKanProm_F/EcoRIK7Kanterm3. This fragment was ligated into EcoRI sites of pJet1.2 *hlpA^Sp^-mTurquoise2*/*mNeonGreen*/*msfYFP/mScarlet-I*/*mKate2* to generate plasmids with APH(3’)-IIIa (accession number V01547.1) located downstream of the *hlpA^Sp^-fp* genes. Fragments containing *catQ* (Cm^R^; accession number CEP28067.1), *erm*(B) (Ery^R^; accession number XMC66049.1) and ANT(9) (Spec^R^; accession number AAC53685.1), along with their native promoters, were amplified from *S. salivarius* strain F4_20 (accession number LN812955.1) (Chaffanel et al. 2015), plasmids pG^+^Host9 (Maguin et al. 1992) and pSet4s (Takamatsu et al. 2001), respectively. Primer pairs EcoRIK7catQ5'/HindK7catQ3', EcoRIEry_F/HindEry_R or EcoRISpecFwdbis/HindSpecRev were used for the amplification. The antibiotic resistance fragments were ligated into EcoRI and HindIII sites of pJet1.2 *hlpA^Sp^-mTurquoise2*/*mNeonGreen*/*msfYFP*/*mScarlet-I*/*mKate2* to construct plasmids with *catQ, erm*(B) or ANT(9) located downstream of the *hlpA^Sp^-fp* genes. All plasmids were verified by sequencing and have been deposited at Addgene (plasmid #206810–206829).

### Construction of *S. thermophilus hlpA^Sp^-fp -ab^R^*

DNA cassettes for *S. thermophilus* transformation were engineered using an overlapping fusion PCR procedure, as described by Mortier-Barriere et al. (2019). Plasmids pJet1.2 *hlpA^Sp^-mTurquoise2*/*mNeonGreen*/*msfYFP*/*mScarlet-I*/*mKate2*-Cm^R^/Ery^R^/Km^R^/Spec^R^ were used as templates for *hlpA^Sp^-fp-*Ab^R^ cassettes for PCR. All 20 different amplicons (PCR hlpA^Sp^-fp-Ab^R^) were amplified using primers hlpA-F-rbs_C, which introduce a ribosome binding site (RBS) for *hlpA^Sp^-fp* translation, and FluoAbR2. For alternative construction, the hlpA_ATG_F primer can be used to initiate at the *hlpA^Sp^* methionine start codon, and FPs can be fused to the C-terminus of a protein of interest, along with Ab^R^ selection.

Homologous fragments including the *hlpA^St^* upstream and the downstream regions were amplified from strain LMG18311 (LMG strain collection) using primer pairs SthHlpANot_1/hlpA-R-rbs_B, and hlpA-down-I/SthHlpA_Apa4, respectively. The two amplicons generated, PCRhlpA_UP and PCRhlpA_DOWN, were each over 1 kb in length to promote homologous recombination. Equimolar amounts of purified PCRhlpA_UP, PCRhlpA_DOWN and PCRhlpA^Sp^-fp-Ab^R^ fragments were fused with overlapping PCRs (see Supplementary Table S5) with outers primers SthHlpANot_1 and SthHlpA_Apa4 (Mortier-Barriere et al. 2019). Five microliters of the resulting amplifications were used for LMG18311 transformation and insertion via double homologous recombination downstream of the *hlpA^St^* gene. Transformants were selected on M17L supplemented with appropriate antibiotics and verified by fluorescence microscopy, PCR and sequencing.

### Construction of fp fusions in *S. salivarius*

All PCR amplicons used as a base for overlapping PCRs are described in the Supplementary Table S5. The *hlpA^Sp^-fp* genes were fused with overlapping PCRs to either a strong constitutive promoter (P_32_) or an aTc-inducible promoter (P*_tet_*) and inserted with a spectinomycin resistance cassette at the permissive tRNA serine locus (*tRNA^Ser^; HSISS4_r00062*) by double homologous recombination. The Up_*tRNA^Ser^*-P_32_ and Spec^R^-Dn_*tRNA^Ser^* fragments were amplified from JM1101 with primers UF_tRNAser/R_P32 or F_spec/DR_tRNAser2, respectively. The *hlpA^Sp^-mTurquoise2*/*mNeonGreen*/*msfYFP*/*mScarlet-I*/*mKate2* fragments were amplified from pJET-*hlpA*-*mTurquoise2*/*mNeonGreen*/*msfYFP*/*mScarlet-I*/*mKate2* with primers F_hlpA_(P32) and R_mTurquoise/R_neonGreen/R_YFP/R_Scarlet/R_mKate2, respectively. To generate the aTc-inducible *hlpA^Sp^-fp* constructs, the Up_*tRNA^Ser^* fragment was amplified from HSISS4 with primers UF_tRNAser/UR_tRNAser, the tetR-P*_tet_* fragment was amplified from VL1048 with primers F_tetR_(ser)/R_tetR and the *hlpA^Sp^-mTurquoise2*/*mNeonGreen*/*mScarlet-I*-Spec^R^-Dn_*tRNA^Ser^* fragments were amplified from VL5852/VL5850/VL5851 with primers F_linker-fp/DR_tRNAser2.

The *ftsZ-fp* genes were fused with overlapping PCRs to the P*_tet_* promoter and inserted by double homologous recombination at either the permissive *tRNA^Ser^* locus or the *tRNA* threonine locus (*tRNA^Thr^; HSISS4_r00061*), along with the *tetR* regulator gene and a spectinomycin or chloramphenicol resistance cassette, respectively. The Up_*tRNA^Ser^*-*tetR*-P*_tet_* fragment was amplified from VL5853 with primers UF_tRNAser/R_tetR, the *ftsZ* cds was amplified from HSISS4 with primers F_ftsZ_(Ptet)/R_ftsZ_linkerfp, and the *mTurquoise2*/*mNeonGreen*/*mScarlet-I*-Spec^R^-Dn_*tRNA^Ser^* fragments were amplified from VL5852/VL5850/VL5851 with primers F_linker-fp/DR_tRNAser2. To transfer the P*_tet_*-*ftsZ-mNeonGreen* cassette to the ectopic *tRNA^Thr^* locus, the Up_*tRNA^Thr^* fragment was amplified from JM1100 with primers UF_tRNAthr/UR_tRNAthr, the *tetR*-P*_tet_*-*ftsZ-mNeonGreen* fragment was amplified from JM1100 with primers F_tetR_(thr)/R_nGreen_(cat), and the Cm^R^-Dn_*tRNA^Thr^* fragment was amplified from JM1100 with primers F_cat/DR_tRNAthr.

The *divIVA-fp* genes were fused by overlapping PCRs to either a strong constitutive promoter (P_32_), or to a strong (P*_xyl1_*) or a mild (P*_xyl2_*) xylose inducible promoter and inserted by double homologous recombination at either the permissive *tRNA^Ser^* locus or the *gor* locus (downstream of *HSISS4_00325*), with the *xylR* regulator gene in the case of P*_xyl1_* and P*_xyl2_*, and with a spectinomycin or erythromycin resistance cassette. The Up_*tRNA^Ser^*-P_32_ fragment was amplified from JM1101 with primers UF_tRNAser/R_P32, the *divIVA* cds was amplified from HSISS4 with primers F_divIVA_(P32)/R_divIVA_linkerfp, and the *mTurquoise2*/*mNeonGreen*/*mScarlet-I*-Spec^R^-Dn_*tRNA^Ser^* fragments were amplified from VL5852/VL5850/VL5851 with primers F_linker-fp/DR_tRNAser2. To construct the xylose- inducible *divIVA*-*mScarlet-I* at *tRNA^Ser^* locus, the Up_*tRNA^Ser^*-*xylR*-P*_xyl1_*/P*_xyl2_* fragments were amplified from JM1015/JM1016 with primers UF_tRNAser/R_pZX9_ATG, and the *divIVA*-*mScarlet-I*-Spec^R^-Dn_*tRNA^Ser^* fragments were amplified from VL5860 with primers F_DivIVA_(Pxyl)/DR_tRNAser2. To transfer the P*_xyl1_*-*divIVA*-*mScarlet-I* cassette to the ectopic *gor* locus, the Up_*gor* and Dn_*gor* fragments were amplified from HSISS4 with primers Fw.Up.gor/Rev.Up.gor and DF_GOR_(ery)/Rev.Dn.gor, the *xylR*-P*_xyl1_*-*divIVA*-*mScarlet-I* fragment was amplified from VL5894 with primers F_xylR_GOR/R_fp_(ery), and the Ery^R^ cassette was amplified from JM1029 with primers Uplox66/DNlox71.

### Construction and selection of integrative shuttle *pNST260+* derivative expressing *hlpA^Sp^-fp*

To generate a broad-host-range integrative vector, we used pNST260+, which carries the erythromycin resistance marker *erm*(B) (Ery^R^) and a temperature-sensitive origin of replication (pWV01^TS^) that permits plasmid maintenance at 30°C but not at 37°C. pNST260+ also contains an *attI* integration site and constitutively expresses the ICE*St1* integrase (*int*) from the P_6_ promoter which mediates site-specific insertion at the 3′ end of the *fda* gene. This locus is highly conserved among firmicutes. Deprived of an excisionase, the integration of pNST260+ is essentially irreversible (Bellanger et al. 2009).

We amplified the pNST260+ backbone by PCR using primers St1attR_For and St1Int_Rev and twelve distinct *hlpA^Sp^-fp*-Ab^R^ inserts were amplified using primers hlpA-F-rbs_C and FluoAbR2 (see Supplementary Tables S2 and S5 for primer sequences and overlapping details, respectively). Each insert was assembled into the pNST260+ backbone using Gibson Assembly Master Mix (New England Biolabs) according to the manufacturer’s instructions. The assemblies were transformed into *E. coli* DH5α and plated on selective medium at 30°C overnight. Because the P_6_ promoter is active in *E. coli* and because *hlpA^Sp^-fp* constitutes an operon with *int*, this step enabled the phenotypic selection of positive fluorescent clones. Plasmid DNA from the candidate clones was purified and verified by sequencing prior to use in subsequent experiments. All plasmids have been deposited at Addgene (plasmid #253369–253380).

After transformation into *S. * (natural) or *S. pyogenes* (electroporation), cells were plated with an antibiotic selective pressure and incubated at 30°C overnight. Four Clones were streaked on fresh plates supplemented with antibiotics and incubated at 30°C overnight. Single colonies were cultured at 37°C overnight and plated on medium with antibiotics. Individual clones were tested for pNST260+ integration in PCR with the primer pair 1967_fda_Rev/2499_2042rc, and check for the loss of free plasmids with the primer pair 2499_2042rc**/**1970_fda_attI_R.

### Plate reader growth and fluorescence measurements

For *S. thermophilus*, growth curves and fluorescence were monitored using clear 96-well plates in an EnSight microplate reader (Perkin Elmer). Overnight cultures were diluted 1:100 in M17L and, after 4 h of incubation at 37°C, adjusted to an OD590nm of 0.02 in M17L. If necessary, M17L was buffered to pH6 or pH8. Each sample (250 µl) was prepared in technical triplicate with the blank (medium only) and, for anaerobic growth, with an overlay of sterile mineral oil (Ahn et al. 2009). The microplates were monitored at 25°C, 37°C, and 45°C during 24 h with measurement taken every 10 min after shaking. Absorbance was measured at a height of 7.5 mm with 50 flashes at 590 nm. Fluorescence measurements were conducted at a height of 9.5 mm with 100 flashes, using the following excitation (ex) and emission (em) settings for each fluorescent protein: 434 nm ex/474 nm em for mTurquoise2; 505 nm ex/520 nm em for mNeonGreen; 513 nm ex/530 nm em for msfYFP; 569 nm ex/594 nm em for mScarlet-I and 588 nm ex/633 nm em for mKate2.

For *S. pneumoniae*, growth curves and fluorescence were monitored using clear 96-well plates in a Spark microplate reader (Tecan). Cultures were incubated for 5 h at 37°C and diluted to a final OD595nm of 0.01 in C+Y and. Each sample (250 µl) was prepared in technical triplicate with the blank (medium only) and monitored at 37°C during 24 h with measurement taken every 10 min after shaking. Absorbance was measured at a height of 7.5 mm with 50 flashes at 595 nm. Fluorescence measurements were conducted at a height of 9.5 mm with 100 flashes, using the following excitation (ex) and emission (em) settings for each fluorescent protein: 430(20) nm ex/485(20) nm em for mTurquoise2; 510(10) nm ex/535(25) nm em for mNeonGreen and msfYFP; 560(20) nm ex/620(20) nm em for mScarlet-I.

### Sample preparation and immunoblotting

*Streptococcus salivarius* cells were overnight-precultured in M17G, diluted the next morning to a final OD∼0.01 in fresh M17G supplemented with aTc and/or xylose, if required, and grown for 6 h to reach mid-exponential phase. Cells were harvested by centrifugation (10 min; 4050 × g). Supernatants were discarded, and the cell pellets were resuspended in 1 ml of cold PBS. To normalize cells quantity across samples, ODs were measured, cells were centrifuged, and pellets were resuspended in specific volumes of buffer A (50 mM Tris-HCl, 50 mM NaCl, 1 mM EDTA, pH 8,0), supplemented with protease inhibitors (Sigma 324 890) and 100 µl Zirconia beads (Biospec, 0 612 731). Cell lysis was performed using a FastPrep homogenizer (MP biomedicals) for 2 cycles of 40 s at 6 m.s^−1^, with 40 s on ice between cycles. Lysates were centrifuged (1 min, 4°C, 13.000 × g) and 100 µl of supernatants were mixed with 20 µl of SDS loading dye. Samples were heated at 95°C for 10 min and cooled down on ice for an additional 10 min.

Crude extracts were separated by SDS-PAGE and blotted on methanol-activated PVDF (polyvinylidenfluoride) membranes (Merck Millipore). Membranes were blocked for 1 h with Tris-buffered saline (20 mM Tris base 150 mM NaCl), 0.05% (v/v) Tween 20 (TBS-T), and 5% (w/v) dry milk, followed by a 1-h incubation with the primary antibodies diluted in TBS-T, containing 5% dry milk. The membranes were washed four times for 5 min each in TBS-T and incubated for 1 h with the secondary antibody diluted in TBS-T with 5% dry milk. After four additional 5 min-washes in TBS-T, the membranes were developed using Super signal West Pico PLUS Chemiluminescent substrate (Protein Biology, 34 577) and visualized with the Fusion Fx Camera (Vilber Lourmat). Primary antibodies included rabbit antisera against mTurquoise2 (anti-GFP, Invitrogen, A6455) and mScarlet-I (anti-DsRed, Takara, 632 496), as well as Mouse monoclonal antibodies against mNeonGreen (ChromoTek, 32F6), all at 1:5 000 dilution. HRP-conjugated Goat anti-rabbit (Abcam, AB205718) and anti-mouse (Promega, W402B) secondary antibodies were used at a 1:5000 and 1:2500 dilutions, respectively.

### Image acquisition

*Streptococcus thermophilus* cells were grown overnight in M17L diluted to an OD of 0.01 in fresh M17L. After 5 h of incubation, 1 µl of culture or mixed cultures were spotted on a 1% agarose pad. Imaging was performed using a Nikon Eclipse Ti-E inverted microscope with a perfect focus system (PFS), pE-100 CoolLED, a Plan Apo λ 100 × 1.45 oil objective (Nikon) and a Hamamatsu ORCA-Flash4.0 V2 C11440-22CU camera (Hamamatsu, Hamamatsu City, Japan). Phase-contrast images were acquired using transmitted light and an exposure time of 20 ms. Epifluorescence microscope laser and detector settings were optimized to discriminate between the different fluorescence signals. Snapshot fluorescence images were acquired with a laser intensity set at 10% and exposure times of 200 ms for mTurquoise2, 100 ms for mNeonGreen, 300 ms for msfYFP and 300 ms for mScarlet-I to minimize photobleaching. Images were exported as 16-bit TIF files. The Nikon Ti filters sets used were: CFP HQ (Ex: 436/20 nm, DM: 455 nm, BA: 480/40 nm), GFPHQ (Ex: 470/40 nm, DM: 495 nm, BA: 525/50 nm), YFPHQ (Ex: 500/20 nm, DM: 515 nm, BA: 535/50 nm), Texas Red (Ex: 560/40 nm, DM: 595 nm, BA: 630/60 nm) as described in Daveri 2023 (Daveri et al. 2023).

*Streptococcus salivarius* cells were grown overnight in CDMG without any inducer and diluted to an OD of 0.01 in fresh CDMG. After 2 h of incubation, when cultures reached an OD of ∼0.1, culture media were supplemented with aTc and/or xylose as appropriate and the incubation continued for an additional 2 h. To image exponentially growing cultures, 1 µl of culture was spotted onto PBS 1% agarose pad. For time-lapse microscopy, 0.7 µl of cells were spotted onto CDMG 1% agarose pad supplemented with aTc and xylose. The pad was maintained at 30°C for the duration of imaging. Pads were placed inside a gene frame (Thermo Fisher Scientific) and sealed with a cover glass as described previously (Gallay et al. 2021). Microscopy was conducted using a Leica DMi8 microscope equipped with a sCMOS DFC9000 GT (Leica) camera and a SOLA light engine (Lumencor’s SPECTRA; 7-channel, solid-state light source for epi-fluorescence imaging) and a ×100/1.40 oil-immersion objective. Phase-contrast images were acquired using transmission light with a 50 ms exposure time. Snapshot fluorescence images were acquired with a 700 ms exposure, while time-lapse laser intensity (50%) and exposure times were optimized to minimize photobleaching with 300 ms for mTurquoise2, 400 ms for mNeonGreen, and 1000 ms for mScarlet-I. The filter sets used included: mTurquoise2 (Ex: 440 nm, BS: 455 nm Chroma, Em: 470/26 nm Chroma ET470/26 m), mNeonGreen (Ex: 490/20 nm, BS: 510 nm, Em: 525/36 nm Chroma ET535/30 m), msfYFP (Ex: 510 nm, BS: 520 nm Chroma 69 008, Em: 535/30 nm Chroma ET535/30 m), mScarlet-I (Ex: 550 nm Chroma ET545/30 X, BS: 595 nm Chroma 69 008, Em: 635/70 nm Chroma ET635/70 m), and mKate2 (Ex: 550 nm Chroma ET575/30, BS: 595 nm Chroma 69 008, Em: 635/70 nm Chroma ET635/70 m). Images acquisition was performed using LasX v.3.4.2.18368 software (Leica).

### Image processing

All microscope images were processed with FIJI v2.14.0 to adjust contrast and brightness. Adjustments were automated to maintain consistent settings across all images within a single panel. Signal-to-noise ratio quantification was performed by segmentation on three independent images for each HlpA-FP fusion. The mean background (noise) value and the mean value intensity (signal) values within segmented cells were extracted for analysis. Deconvolution of time-lapse images was performed with the Huygens v.17.10.0p4 (SVI) software. Default settings were applied, and the number of iterations was optimized for mTurquoise2 (9x), mNeonGreen (9x), and mScarlet-I (12x). Time-lapse images were corrected for drift to mount the time-lapse movie.

## Supplementary Material

uqag006_Supplemental_Files
