# Supplementary material for: A modular genetic toolbox for precise gene regulation and multi-color imaging in streptococci
Source: Microlife. 2026 Feb 26;7:uqag006. doi: 10.1093/femsml/uqag006 (PMC12981332; doi:10.1093/femsml/uqag006)
Supplement: uqag006_Supplemental_Files [file uqag006_supplemental_files.zip › Mignolet_et_al_fluo_SI_Rev.docx]

## Supplemental Information

##
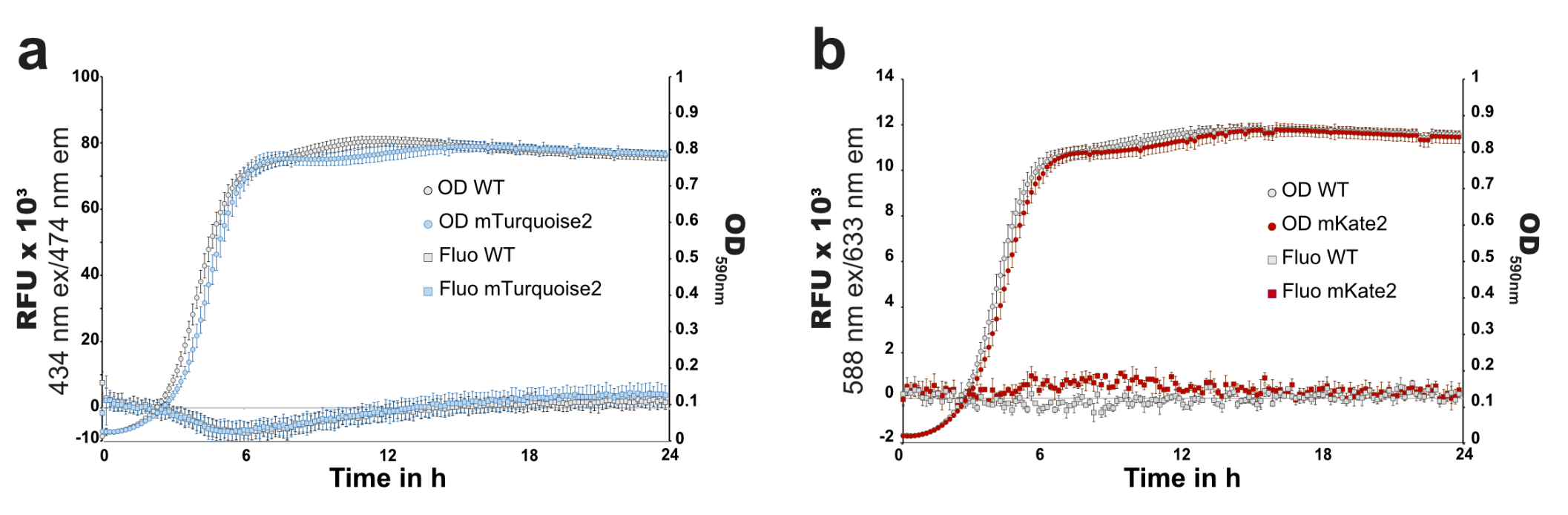


## Supplementary Figure S1. Growth curves and fluorescence quantifications of *S. thermophilus* *hlpA^Sp^-mTurquoise2* and *hlpA^Sp^-mKate2* strains.

Optical density (OD_590nm_, round symbols) and fluorescence measurements, expressed in Relative Fluorescence Units (RFUs, square symbols), of *S. thermophilus* LMG18311 and its derivative strains grown in M17L medium without antibiotics over 24 hours post-dilution. Each LMG18311 derivative is represented by its distinct colored plot symbol, while the absence of fluorescence in the parental LMG18311 strain is plotted in grey. RFU measurements were taken at 434 nm ex/474 nm em for mTurquoise2 (**a**) and 588 nm ex/633 nm em for mKate2 (**b**). All experiments were performed in triplicate.


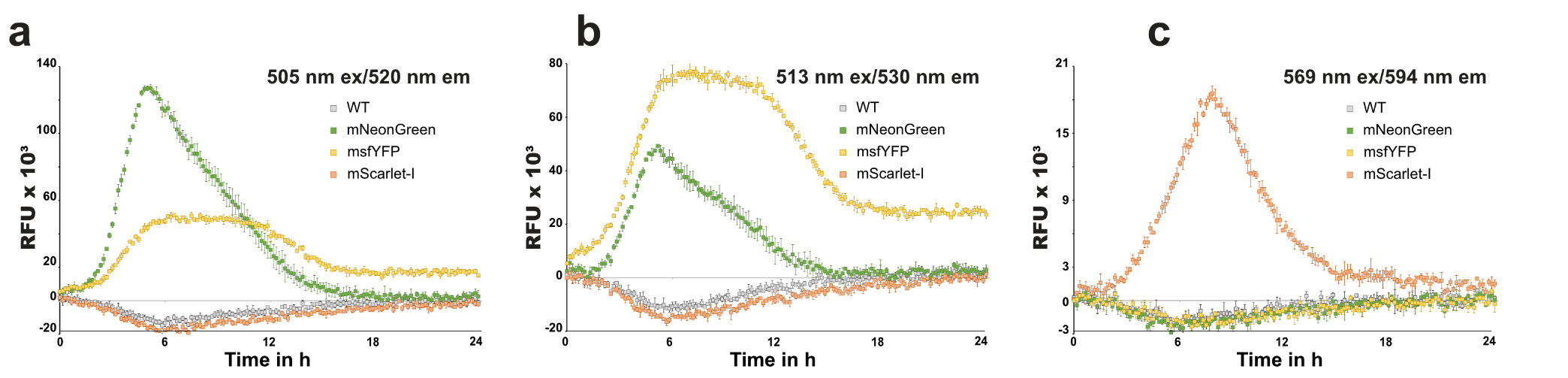


## Supplementary Figure S2. Signal bleed-through for HlpA^Sp^-FP in *S. thermophilus*.

Comparative RFU values of the 3 fluorescent protein fusions HlpA^Sp^-mNeonGreen, HlpA^Sp^-msfYFP and HlpA^Sp^-mScarlet-I measured at each of their specific excitation/emission wavelengths. Each engineered LMG18311 strain is represented by its distinct colored square symbol, while the absence of fluorescence of the parental LMG18311 strain is plotted in grey. RFU measurements were taken at 505 nm ex/520 nm em (**a**), 513 nm ex/530 nm em (**b**), and 569 nm ex/594 nm em (**c**). All experiments were performed in triplicate.

##
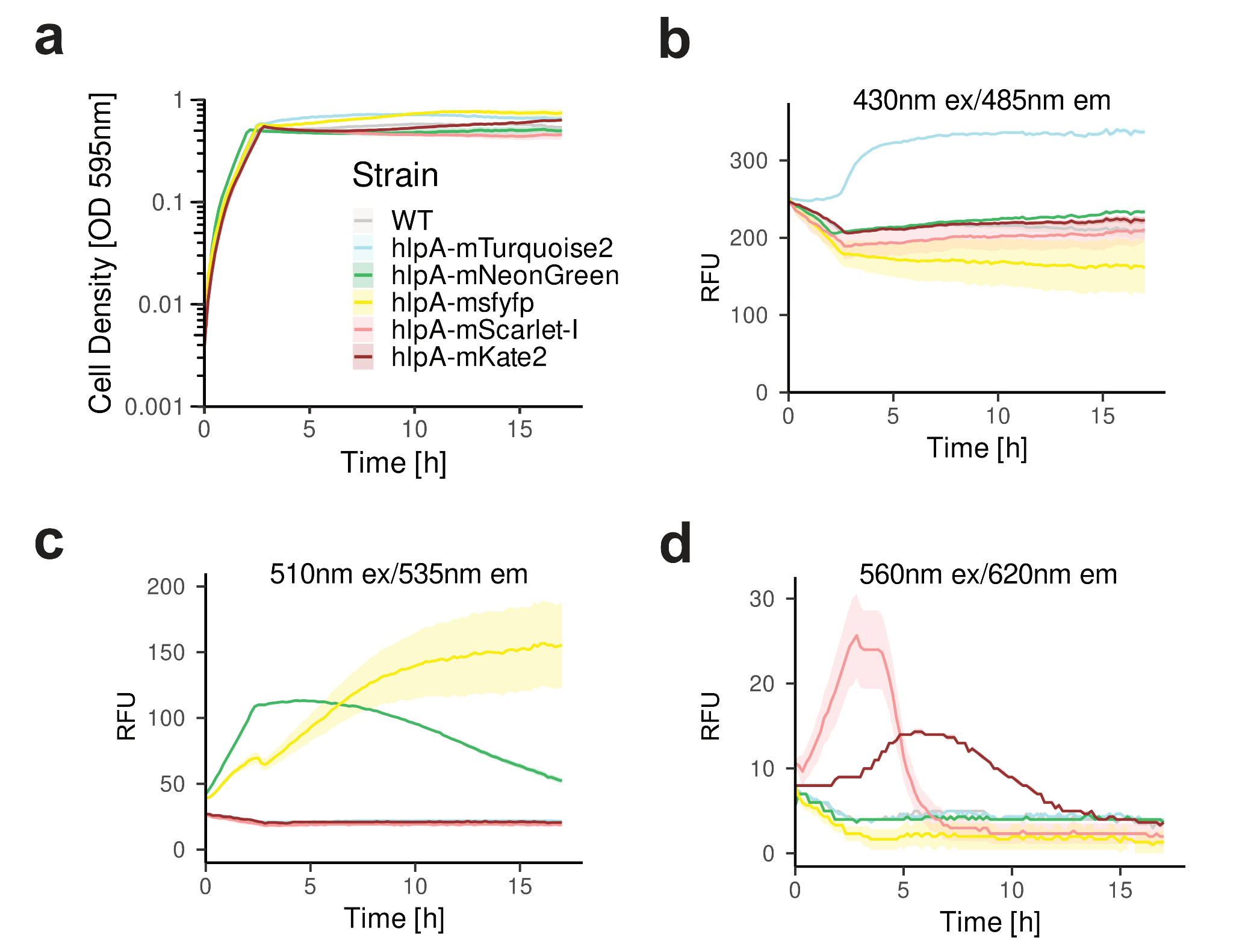


## Supplementary Figure S3. Growth curves and fluorescence quantifications for HlpA^Sp^-FP in *S. pneumoniae*.

Comparative RFU values of the 5 fluorescent protein fusions HlpA^Sp^-mTurquoise2, HlpA^Sp^-mNeonGreen, HlpA^Sp^-msfYFP, HlpA^Sp^-mScarlet-I and HlpA^Sp^-mKate2 measured at each of their specific excitation/emission wavelengths. Each engineered *S. pneumoniae* D39V strain is represented by its native color, while the D39V parental strain is plotted in grey. OD595nm (**a**) and RFU measurements were taken at 430 nm ex/485 nm em (**b**), 510 nm ex/535 nm em (**c**), and 560 nm ex/620 nm em (**d**). All experiments were performed in triplicate. Experimental values represent the averages (with SEM depicted as ribbons) of three independent growth curves.

**
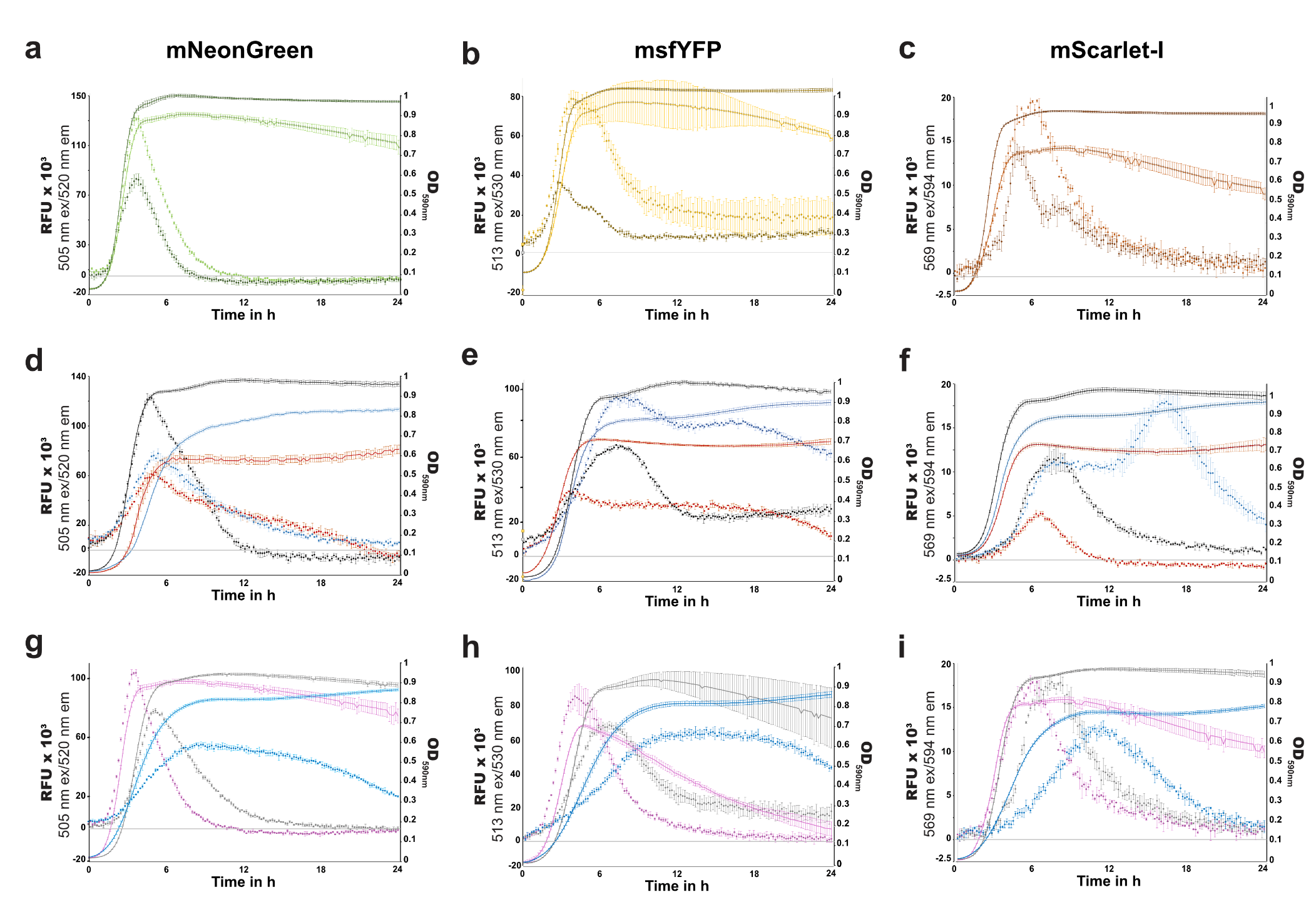
**

**Supplementary Figure S4. Fluorescence signals of *S. thermophilus* *hlpA^Sp^-fp* under varying oxygen availability, pH, and temperature during growth**

Fluorescence measurements of the growth of LMG18311 *hlpA^Sp^-mNeonGreen* (**a**, **d**, **g**), LMG18311 *hlpA^Sp^-msfYFP* (**b**, **e**, **h**) and LMG18311 *hlpA^Sp^-mScarlet-I* (**c**, **f**, **i**) in M17L medium. Growth curves are assessed via culture turbidity at 590 nm (curves) and fluorescence measurements are expressed in Relative Fluorescence Units signal (RFUs, round symbols). RFU measurements (subtracted by signal in M17L) were taken at 505 nm ex/520 nm em (mNeonGreen), 513 nm ex/530 nm em (msfYFP), and 569 nm ex/594 nm em (mScarlet-I). The effects of oxygen conditions are shown in panels **a**, **b**, **c**. Each engineered strain is represented by its native color, light shaded in aerobic conditions and dark shaded in oxygen-limiting conditions. The effects of pH levels are shown in **d**, **e**, **f**. pH 6 is red-colored; pH7 is black-colored and pH 8 is dark blue-colored. The effects of temperatures are shown in **g**, **h**, **i**. 25°C is light blue-colored; 37°C is gray-colored and 45°C is pink-colored. Data points are the means from triplicate assays with error bars indicating the mean ± standard deviation.

**
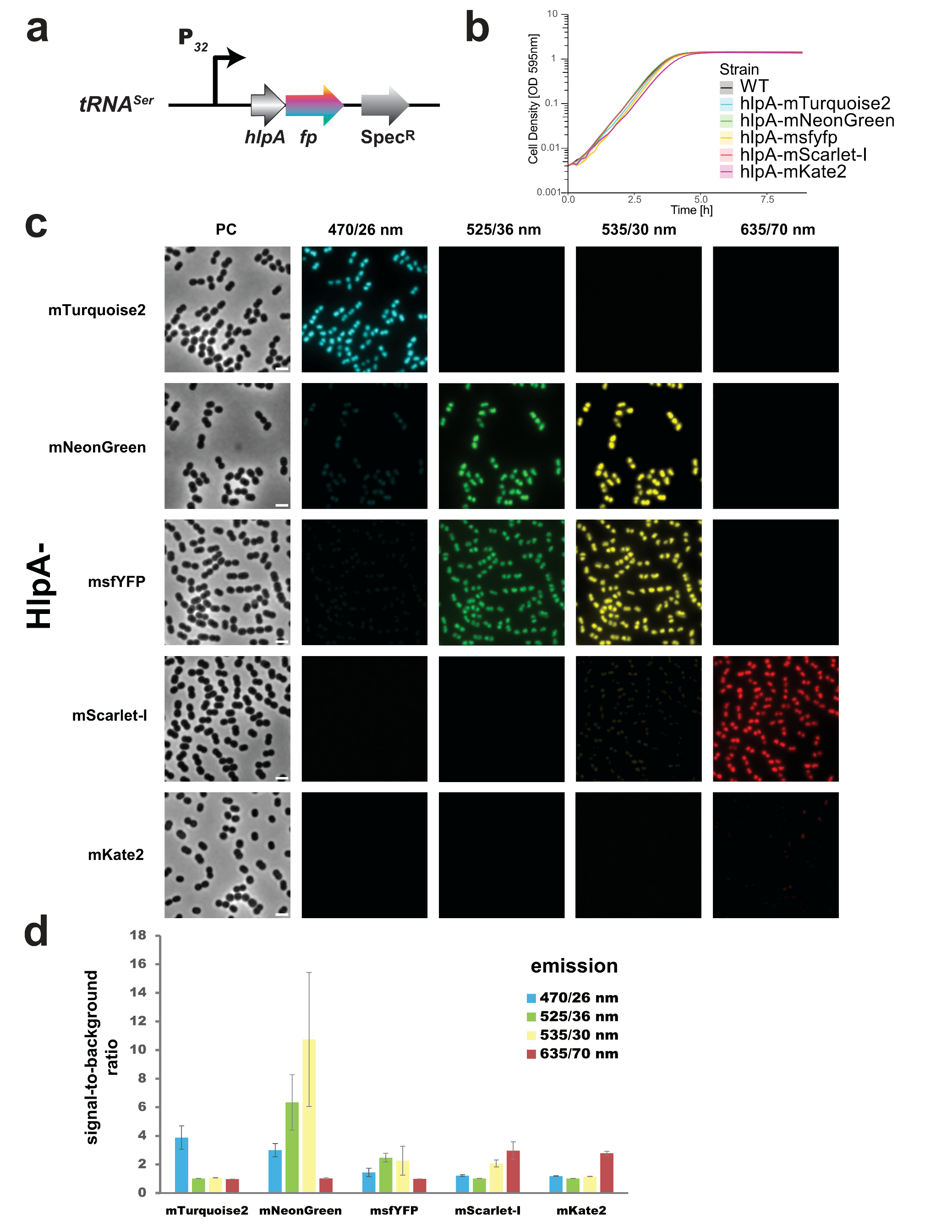
**

**Supplementary Figure S5. Fluorescence channel specificity for 5 FPs in *S. salivarius***

(**a**) Scheme of the genomic topology of the constitutive P*_32_* controlling the *hlpA-fp* integrated at the *tRNA^Ser^* locus. The Spec^R^ gene is used to select chromosomal integration of *hlpA-fp* constructs.

(**b**) Growth curves (OD_595nm_ is shown with a logarithmic scale and expressed over time of *hlpA-fp* constructs. Experimental values represent the averages (with SEM depicted as ribbons) of three independent growth curves.

(**c**) Fluorescent protein tags of the HlpA^Sp^ nucleoid-associated factor were observed under microscope for single-cell analyses. Phase contrast (PC) and fluorescence recorded in the four channels to assess bleed-through are displayed for mTurquoise2, mNeonGreen, msfYFP, mScarlet-I and mKate2 fluorescent tags. Acquisition times for PC were of 50ms and identical (700ms) in all fluorescent channels. The same brightness/contrast parameters were applied to the fluorescence images to evaluate the bleed-through. The scale bar equals 2 μm.

(**d**) Quantification histogram of signal-to-noise ratio for five FPs in the 4 cognate emission channels. Images were segmented and analyzed with the Fiji software to elicit the mean fluorescence intensity per cell. Noise was estimated as the mean value of the background (image area excluding the cell bodies). Experimental values represent the averages (with SEM) of three independent images.

**
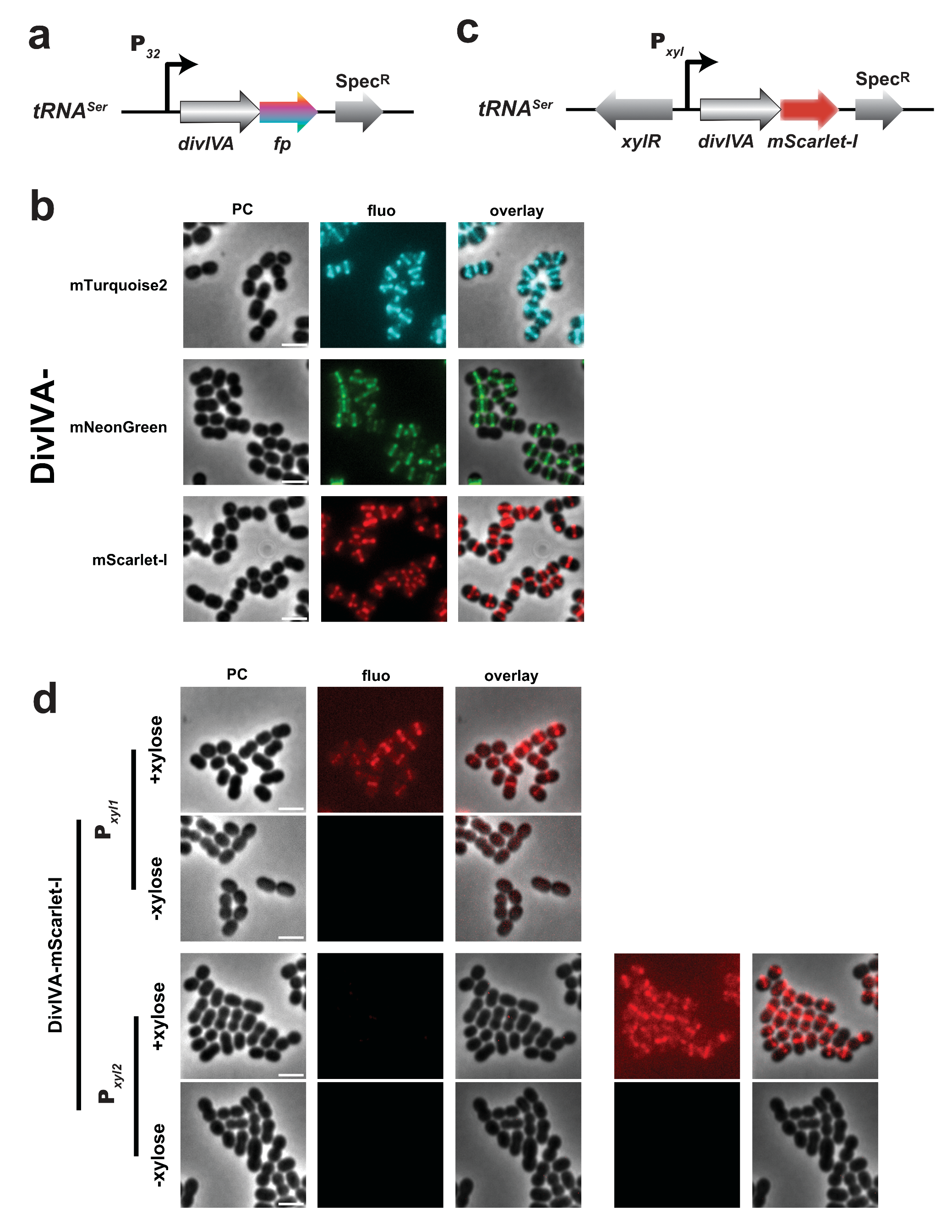
**

**Supplementary Figure S6. DivIVA subcellular localization in *S. salivarius***

(**a**) Scheme of the genomic topology of the constitutive P*_32_* controlling the *divIVA-fp* integrated at the *tRNA^Ser^* locus. The Spec^R^ gene is used to select chromosomal integration of *divIVA-fp* constructs.

(**b**) Single-cell imaging of constitutively-produced tagged DivIVA. mTurquoise2, mNeonGreen and mScarlet-I fusion mutants were imaged in mid-exponential phase. Phase contrast (PC), fluorescence recorded in the cognate channel, and overlay images are depicted. The scale bar equals 2 μm.

(**c**) Scheme of the genomic topology of the P*_xyl_*-inducible *divIVA-mScarlet-I* integrated at the *tRNA^Ser^* locus. The *xylR* repressor genes, with its own promoter (not depicted here), points in the opposite orientation compared to the strong P*_xyl1_* or mild P*_xyl2_* inducible promoter. The Spec^R^ gene is used to select chromosomal integration of *divIVA-fp* constructs.

(**d**) Single-cell imaging of xylose-inducible tagged DivIVA. mScarlet-I fusion mutants were incubated with xylose for 2 hours before imaging. Phase contrast (PC), fluorescence, and overlay images are depicted. The same brightness/contrast parameters were applied to the fluorescence images to qualitatively compare the intensities between the P*_xyl1_* or P*_xyl2_* expression system (left panel). On the bottom right, the brightness/contrast parameters were adjusted to visualize the fluorescent signal in the P*_xyl2_* expression system. The scale bar equals 2 μm.

**
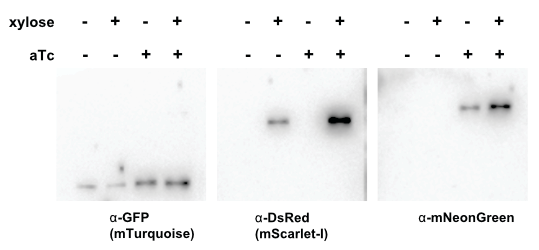
**

**Supplementary Figure S7. Steady-state production level of fluorescent protein fusions**

Full-scan image of immunoblot showing the HlpA-mTurquoise2, FtsZ-mNeonGreen, and DivIVA-mScarlet-I steady-state levels. Cells were grown in M17G supplemented with (+) or without (-) inducing molecules (aTc and/or xylose). The mTurquoise2, mScarlet-I and mNeonGreen fusions were detected with an anti-GFP, anti-DsRed and anti-mNeonGreen primary antibodies, respectively.

**
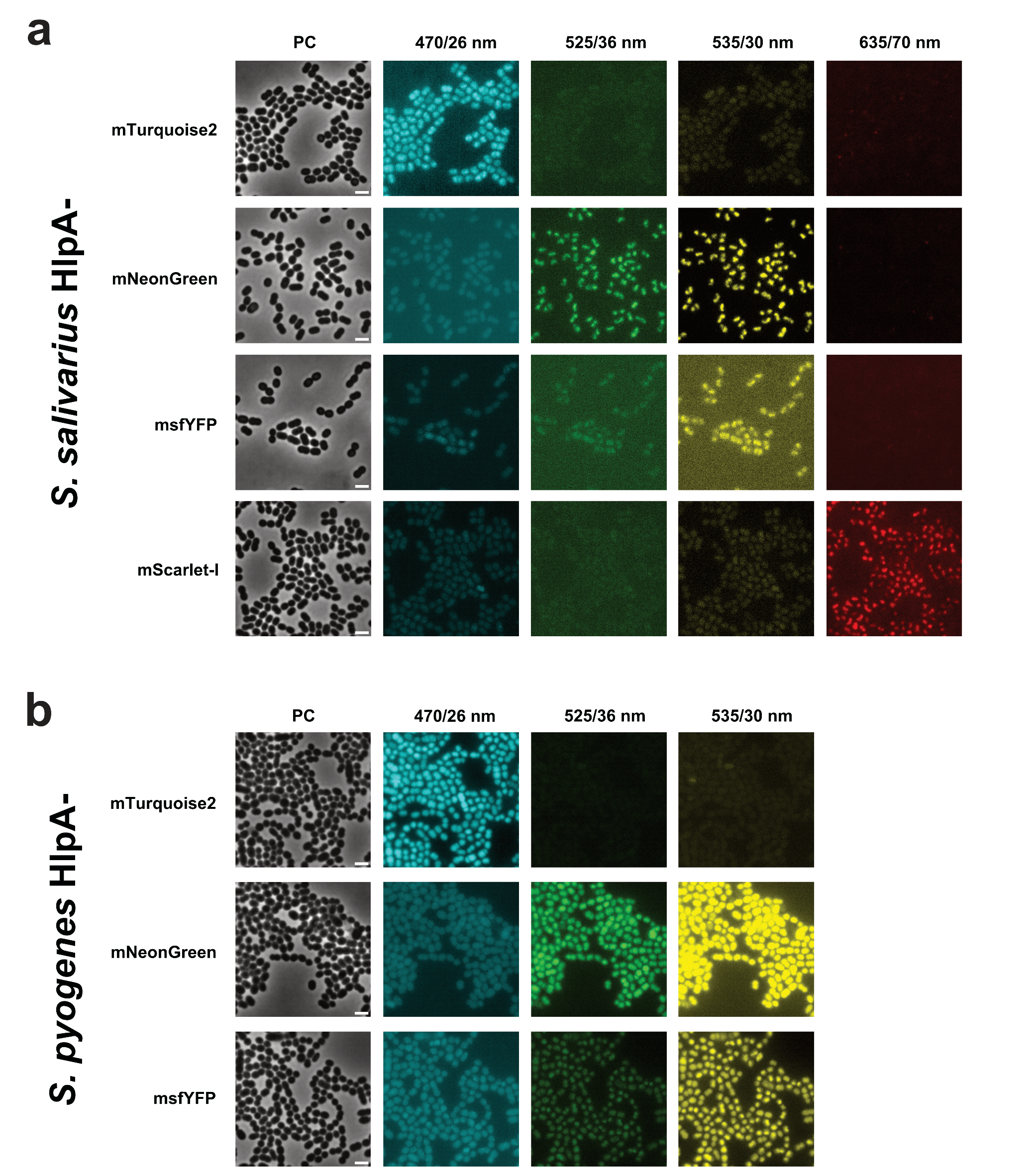
Supplementary Figure S8. Fluorescence channel specificity for4 integrative fluorescent tools in *S. salivarius* and *S. pyogenes***

(**a**) Fluorescent protein tags of the HlpA^Sp^ nucleoid-associated factor in *S. salivarius* were observed under microscope for single-cell analyses. Phase contrast (PC) and fluorescence recorded in the four channels to assess bleed-through are displayed for mTurquoise2, mNeonGreen, msfYFP and mScarlet-I fluorescent tags. The same brightness/contrast parameters were applied to the fluorescence images to evaluate the bleed-through. The scale bar equals 2 μm.

(**b**) Fluorescent protein tags of the HlpA^Sp^ nucleoid-associated factor in *S. pyogenes* were observed under microscope for single-cell analyses. Phase contrast (PC) and fluorescence recorded in the three channels to assess bleed-through are displayed for mTurquoise2, mNeonGreen and msfYFP fluorescent tags. The same brightness/contrast parameters were applied to the fluorescence images to evaluate the bleed-through. The scale bar equals 2 μm.

**Supplementary Movie S1**

Time-lapse movie of the multi-labeled strain HlpA^Sp^-mTurquoise2 FtsZ-mNeonGreen DivIVA-mScarlet-I. Cells were incubated with aTc and xylose for 2 hours before imaging. The inducing molecules were supplemented in the agarose pad. Phase contrast (PC), fluorescence recorded in the three cognate channels, and overlay images are depicted. Individual fluorescent images were deconvoluted with the Huygens software and assembled with Fiji. The scale bar equals 2 μm.

## Supplementary Table S1 Plasmids

|  | Characteristics | Reference / #plasmid |  |
| --- | --- | --- | --- |
| pJet1.2 | | blunt positive-selection cloning vector *bla* | Invitrogen |
| pJethlpAmKate2Cm | pJet1.2 *hlpA^Sp^-mKate2 catQ* | This work / #206810 |  |
| pJethlpAmKate2Ery | pJet1.2 *hlpA^Sp^-mKate2 erm*(B) | This work / #206811 |  |
| pJethlpAmKate2Km | pJet1.2 *hlpA^Sp^-mKate2* APH(3’)-IIIa | This work / #206812 |  |
| pJethlpAmKate2Spc | pJet1.2 *hlpA^Sp^-mKate2* ANT(9*)* | This work / #206813 |  |
| pJethlpAmNeonGreenCm | pJet1.2 *hlpA^Sp^-mNeonGreen catQ* | This work / #206814 |  |
| pJethlpAmNeonGreenEry | pJet1.2 *hlpA^Sp^-mNeonGreen erm*(B) | This work / #206815 |  |
| pJethlpAmNeonGreenKm | pJet1.2 *hlpA^Sp^-mNeonGreen* APH(3’)-IIIa | This work / #206816 |  |
| pJethlpAmNeonGreenSpc | pJet1.2 *hlpA^Sp^-mNeonGreen* ANT(9) | This work / #206817 |  |
| pJethlpAmScarletCm | pJet1.2 *hlpA^Sp^-mScarlet-I catQ* | This work / #206818 |  |
| pJethlpAmScarletEry | pJet1.2 *hlpA^Sp^-mScarlet-I erm*(B) | This work / #206819 |  |
| pJethlpAmScarletKm | pJet1.2 *hlpA^Sp^-mScarlet-I* APH(3’)-IIIa | This work / #206820 |  |
| pJethlpAmScarletSpc | | pJet1.2 *hlpA^Sp^-mScarlet-I* ANT(9) | This work / #206821 |
| pJethlpAmTurquoise2Cm | pJet1.2 *hlpA^Sp^-mTurquoise2 catQ* | This work / #206822 |  |
| pJethlpAmTurquoise2Ery | | pJet1.2 *hlpA^Sp^-mTurquoise2 erm*(B) | This work / #206823 |
| pJethlpAmTurquoise2Km | pJet1.2 *hlpA^Sp^-mTurquoise2* APH(3’)-IIIa | This work / #206824 |  |
| pJethlpAmTurquoise2Spc | | pJet1.2 *hlpA^Sp^-mTurquoise2* ANT(9) | This work / #206825 |
| pJethlpAYFPCm | pJet1.2 *hlpA^Sp^-msfYFP catQ* | This work / #206826 |  |
| pJethlpAYFPEry | | pJet1.2 *hlpA^Sp^-msfYFP erm*(B) | This work / #206827 |
| pJethlpAYFPKm | pJet1.2 *hlpA^Sp^-msfYFP* APH(3’)-IIIa | This work / #206828 |  |
| pJethlpAYFPSpc | | pJet1.2 *hlpA^Sp^-msfYFP* ANT(9) | This work / #206829 |
|  | |  |  |
| pNST260+ | | Integrative Ery^R^ pG+host9-derived plasmid | (Bellanger et al. 2009) |
| pNST260+mTurquoise2Km | | pNST260+*hlpA^Sp^-mTurquoise2* APH(3’)-IIIa | This work / #253369 |
| pNST260+mNeonGreenKm | | pNST260+*hlpA^Sp^-mNeonGreen* APH(3’)-IIIa | This work / #253370 |
| pNST260+msfYFPKm | | pNST260+*hlpA^Sp^-msfYFP* APH(3’)-IIIa | This work / #253371 |
| pNST260+mScarletKm | | pNST260+*hlpA^Sp^-mScarlet-I* APH(3’)-IIIa | This work / #253372 |
| pNST260+mTurquoise2Cm | | pNST260+*hlpA^Sp^-mTurquoise2 catQ* | This work / #253373 |
| pNST260+mNeonGreenCm | | pNST260+*hlpA^Sp^-mNeonGreen catQ* | This work / #253374 |
| pNST260+msfYFPCm | | pNST260+*hlpA^Sp^-msfYFP catQ* | This work / #253375 |
| pNST260+mScarletCm | | pNST260+*hlpA^Sp^-mScarlet-I catQ* | This work / #253376 |
| pNST260+mTurquoise2Spc | | pNST260+*hlpA^Sp^-mTurquoise2* ANT(9) | This work / #253377 |
| pNST260+mNeonGreenSpc | | pNST260+*hlpA^Sp^-mNeonGreen* ANT(9) | This work / #253378 |
| pNST260+msfYFPSpc | | pNST260+*hlpA^Sp^-msfYFP* ANT(9) | This work / #253379 |
| pNST260+mScarletSpc | | pNST260+*hlpA^Sp^-mScarlet-I* ANT(9) | This work / #253380 |

## Supplementary Table S2 Oligonucleotides

| Names | Sequences |
| --- | --- |
| hlpA-up-F | 5’-AACAAGTCAGCCACCTGTAG-3’ |
| hlpA-up-R | 5’-**TGATCCTTTAGCTGCAGCTT**CTCCACC-3’ |
| cam-hlpA-down-F | 5’-**TAAGAATTCTAATGAGCACT**AGTAGG-3’ |
| cam-hlpA-down-R | 5’-CGTGGCTGACGATAATGAGG-3’ |
| mTurquoise2-F | 5’-**AAGCTGCAGCTAAAGGATCA**GTGAGCAAGGGCGAGGAGCTG-3’ |
| mTurquoise2-R | 5’-**AGTGCTCATTAGAATTCTTA**TTACTTGTACAGCTCGTCCATGCCGAG-3’ |
| mNeon-F | 5’-**AAGCTGCAGCTAAAGGATCA**GTCTCTAAAGGTGAAGAAGATAATATGG-3’ |
| mNeon-R | 5’-**AGTGCTCATTAGAATTCTTA**TTATTTATACAATTCATCCATACCC-3’ |
| hlpA_LB_F | 5’-catggcaaacaaacaagatttgatcgctaaag-3’ |
| Cm-int-R | 5’-AAAGTCGTTTGTTGGTTCAAATAATGAT-3’ |
| EcoRIKanProm_F | 5’-CCGAATTCACGTTAACAACCGGTACCTC-3’ |
| EcoRIK7Kanterm3 | 5’-CGGGAAATTTGCAGAGCAGGTAGCTATTC-3’ |
| EcoRIK7catQ5' | 5’-CCGAATTCCGTATTGGTGGAATAACACG-3’ |
| HindK7catQ3' | 5’-CTGAAGCTTGCTCTGCTTAGGATAAATAGC-3’ |
| EcoRIEry_F | 5’-CCGAATTCCAAACTTAAGAGTGTGTTGATAGTGC-3’ |
| HindEry_R | 5’-CTGAAGCTTGGACCTCTTTAGCTCCTTGG-3’ |
| EcoRISpecFwdbis | 5’-CCGAATTCCGAAGTTATCGTAACGTGAC-3’ |
| HindSpecRev | 5’-CTGAAGCTTCCAATTAGAATGAATATTTCCC-3’ |
| hlpA-F-rbs_C | 5’-GCATGCTGGAGGAATCATTAACATGGCAAACAAACAAGATTTGA-3’ |
| hlpA_ATG_F | 5’-ATGGCAAACAAACAAGATTTG-3’ |
| FluoAbR2 | 5’-TGGATGCTATTAACCCTGAACTTTCTT-3’ |
| SthHlpANot_1 | 5’-GGGCGGCCGCGTCAGGTGAGTAAGGTATG-3’ |
| hlpA-R-rbs_B | 5’-GTTAATGATTCCTCCAGCATGCGGGATTAACATTATTTGACTGCGT-3’ |
| hlpA-down-I | 5’-GTTCAGGGTTAATAGCATCCACCTAAGCTTTTAGGGGTTTTGA-3’ |
| SthHlpA_Apa4 | 5’-CGGGGCCCGTCAGAAGCTTATACCTTATCC-3’ |
| UF_tRNAser | 5’-CAAGATTAACCATGACCTTC-3’ |
| DR_tRNAser2 | 5’-TTGGATAAGGTCTTGACTTC-3’ |
| R_P32 | 5’-**CATCTAAATTCCTCCTCTAG**-3’ |
| F_spec | 5’-**TAATAAGGCCGGCCAATAAA**-3’ |
| F_hlpA_(P32) | 5’-**CTAGAGGAGGAATTTAGATG**gcaaacaaacaagatttga-3’ |
| R_mKate2 | 5’-**TTTATTGGCCGGCCTTAtta**acggtgtcccaatttac-3’ |
| R_neonGreen | 5’-**TTTATTGGCCGGCCTTAtta**tttatacaattcatcc-3’ |
| R_Scarlet | 5’-**TTTATTGGCCGGCCTTAtta**tttatatagttcgtcc-3’ |
| R_mTurquoise | 5’-**TTTATTGGCCGGCCTTATTA**CTTGTACAGCTCGTCC-3’ |
| R_YFP | 5’-**TTTATTGGCCGGCCTTATTA**TTTATAAAGTTCGTCC-3’ |
| St1attR_For | 5’-**TCAGGGTTAATAGCATCCA**GTGTGGCAAATCTGTGGCAA-3’ |
| St1Int_Rev | 5’-**CATGTTAATGATTCCTCCAGC**TTATTGTGTAAGAGTATCAAGT-3’ |
| pNST_Rbs-hlpA | 5’-**GCTGGAGGAATCATTAAC**ATGGCAAACAAACAAGATTTGA-3’ |
| 1967_fda_Rev | 5’-CTTGGTGTTGCTAAAGTT-3’ |
| 2499_2042rc | 5’-AAGAAAGTTCAGGGTTAATAGCATCCA-3’ |
| 1970_fda_attI_R | 5’-GATGAAATTCACATCATC-3’ |
| UR_tRNAser | 5’-**AGTAATTAAAAAGAAGATGG**-3’ |
| F_tetR_(ser) | 5’-**CCATCTTCTTTTTAATTACT**CTATTCATTGTTTTGCATGC-3’ |
| R_Ptet | 5’-**CATTATTTTTCCTCCTTATTT**-3’ |
| F_hlpA_(Ptet) | 5’-**AAATAAGGAGGAAAAATAATG**gcaaacaaacaagatttga-3’ |
| F_linker-fp | 5’-**AGAGGATCTGGTGGAGAAG**-3’ |
| F_ftsZ_(Ptet) | 5’-**AAATAAGGAGGAAAAATAATG**agtttttcatttgatagc-3’ |
| R_ftsZ_linkerfp | 5’-**CTTCTCCACCAGATCCTCT**acgatttttaaagaatggtg-3’ |
| F_divIVA_(P32) | 5’-**CTAGAGGAGGAATTTAGATG**gctattacagcacttg-3’ |
| R_divIVA_linkerfp | 5’-**CTTCTCCACCAGATCCTCT**ttcgctaatattcaatttaaac-3’ |
| R_pZX9_ATG | 5’-**CATATTTACCTCCTTTGATTTA**-3’ |
| F_DivIVA_(Pxyl) | 5’-**TAAATCAAAGGAGGTAAATatg**gctattacagcacttg-3’ |
| UF_tRNAthr | 5’-TGTCAAAGGATTAGGAAAAC-3’ |
| UR_tRNAthr | 5’-**TTGATTTATACCTCTCAATTT**-3’ |
| F_cat | 5’-**tagaccccggggatcctc**-3’ |
| DR_tRNAthr | 5’-aaggagaaaattatgtacac-3’ |
| F_tetR_(thr) | 5’-**AAATTGAGAGGTATAAATCAA**CTATTCATTGTTTTGCATGC-3’ |
| R_nGreen_(cat) | 5’-**gaggatccccggggtcta**ttatttatacaattcatcc-3’ |
| Fw.Up.gor | 5’-GGTGTTAAATTGACTGAAAAAG-3’ |
| Rev.Up.gor | 5’-**TGAGTGAATGGTTTCAATTG**-3’ |
| DF_GOR_(ery) | 5’-**TACATTCCCTTTAGTAACGTGAA**aacgggttcagaagaatttg-3’ |
| Rev.Dn.gor | 5’-GCTCAAACATTTTCTAAGATTAC-3’ |
| F_xylR_GOR | 5’-**caattgaaaccattcactca**tctagattatatatgatatgatc-3’ |
| R_fp_(ery) | 5’-**CCTTATGGGATTTATCTTCCTTA**AAGAATCTTGCTTGGCAAGG-3’ |
| Uplox66 | 5’-**TAAGGAAGATAAATCCCATAAGG**-3’ |
| DNlox71 | 5’-**TTCACGTTACTAAAGGGAATGTA**-3’ |

Overlapping sequences are highlighted in bold.

**Supplementary Table S3 Fluorescent tag references and properties**

| **Fluorescent tag** | **FPbase ID** | **Patent** | **Excitation** | **Emission** |
| --- | --- | --- | --- | --- |
| mTurquoise2 | 7AV5G |  | 434 | 474 |
| mNeonGreen | ZRKRV | US 10,221,221B2 | 506 | 517 |
| msfYFP | EFRBB | US 7,271,241B2 | 514 | 529 |
| mScarlet-I | 6VVTK | WO2022253948A1 | 569 | 593 |
| mKate2 | DBBO8 | US 7,638,615B1 | 588 | 633 |

## Supplementary Table S4 Strains

|  | | Characteristics | Reference/source |  |
| --- | --- | --- | --- | --- |
| *Escherichia coli* | | |  |  |
| DH5α | | *supE hsd-5 thi (lac-proAB) F (traD6 proAB lacIq lacZ M15) repA, derivative of strain TG1 (56) repA, derivative of strain JM101* | Invitrogen |  |
| *Streptococcus thermophilus* | | |  |  |
| LMG18311 | | Wild-type strain | BCCM/LMG, strain collection |  |
| SC00501 | | LMG18311 *hlpA-hlpA^Sp^-mTurquoise2-*Ery^R^ | This work |  |
| SC00502 | | LMG18311 *hlpA-hlpA^Sp^-mNeonGreen-*Ery^R^ | This work |  |
| SC00503 | | LMG18311 *hlpA-hlpA^Sp^-msfYFP-*Ery^R^ | This work |  |
| SC00504 | | LMG18311 *hlpA*-*hlpA^Sp^-mScarlet-I-*Ery^R^ | This work |  |
| SC00505 | | LMG18311 *hlpA-hlpA^Sp^-mKate2-*Ery^R^ | This work |  |
| *Streptococcus salivarius* | | |  |  |
| HSISS4 | | Wild-type gastro-intestinal tract isolate | (Van den Bogert et al. 2014) |  |
| JM1101 | | HSISS4 *tRNA^Ser^::*P*_32_-scuR*-Spec^R^ | (Mignolet et al. 2019) |  |
| JM1015 | | HSISS4 *tRNA^Ser^::*P*xyl1-comR-*Spec^R^ | (Mignolet et al. 2018) |  |
| JM1016 | | HSISS4 *tRNA^Ser^::*P*xyl2-comR-*Spec^R^ | (Mignolet et al. 2018) |  |
| JM1100 | | HSISS4 *tRNA^Thr^::*P*_sptA_-luxAB*-Cm^R^ | (Mignolet et al. 2019) |  |
| JM1029 | | HSISS4 *tRNA^Thr^::*P*_comS_-luxAB*-Cm^R^ Δ*comRS::*Ery^R^ | (Mignolet et al. 2018) |  |
| VL5850 | | HSISS4 *tRNA^Ser^::*P*_32_-hlpA^Sp^-mNeonGreen-*Spec^R^ | This work |  |
| VL5851 | | HSISS4 *tRNA^Ser^::*P*_32_-hlpA^Sp^-mScarlet-I-*Spec^R^ | This work |  |
| VL5852 | | HSISS4 *tRNA^Ser^::*P*_32_-hlpA^Sp^-mTurquoise2-*Spec^R^ | This work |  |
| VL5942 | | HSISS4 *tRNA^Ser^::*P*_32_-hlpA^Sp^-mKate2-*Spec^R^ | This work |  |
| VL5943 | | HSISS4 *tRNA^Ser^::*P*_32_-hlpA^Sp^-msfYFP-*Spec^R^ | This work |  |
| VL5853 | | HSISS4 *tRNA^Ser^::*P*_tet_-hlpA^Sp^-mNeonGreen-*Spec^R^ | This work |  |
| VL5854 | | HSISS4 *tRNA^Ser^::*P*_tet_-hlpA^Sp^-mScarlet-I-*Spec^R^ | This work |  |
| VL5855 | | HSISS4 *tRNA^Ser^::*P*_tet_-hlpA^Sp^-mTurquoise2-*Spec^R^ | This work |  |
| VL5856 | | HSISS4 *tRNA^Ser^::*P*_tet_-ftsZ-mNeonGreen-*Spec^R^ | This work |  |
| VL5857 | | HSISS4 *tRNA^Ser^::*P*_tet_-ftsZ-mScarlet-I-*Spec^R^ | This work |  |
| VL5858 | | HSISS4 *tRNA^Ser^::*P*_tet_-ftsZ-mTurquoise2-*Spec^R^ | This work |  |
| VL5859 | | HSISS4 *tRNA^Ser^::*P*_32_-divIVA-mNeonGreen-*Spec^R^ | This work |  |
| VL5860 | | HSISS4 *tRNA^Ser^::*P*_32_-divIVA-mScarlet-I-*Spec^R^ | This work |  |
| VL5861 | | HSISS4 *tRNA^Ser^::*P*_32_-divIVA-mTurquoise2-*Spec^R^ | This work |  |
| VL5894 | | HSISS4 *tRNA^Ser^::*P*_xyl1_-divIVA-mScarlet-I-*Spec^R^ | This work |  |
| VL5895 | | HSISS4 *tRNA^Ser^::*P*_xyl2_-divIVA-mScarlet-I-*Spec^R^ | This work |  |
| VL5930 | | HSISS4 *tRNA^Thr^::*P*_tet_-ftsZ-mNeonGreen-*Cm^R^ | This work |  |
| VL5932 | | HSISS4 *gor::*P*_xyl1_-divIVA -mScarlet-I-*Ery^R^ | This work |  |
| VL5931 | | VL5852 *tRNA^Thr^::*P*_tet_-ftsZ-mNeonGreen-*Cm^R^ | This work |  |
| VL5940 | | VL5931 *gor::*P*_xyl1_-divIVA-mScarlet-I-*Ery^R^ | This work |  |
| VL8249 | | HSISS4 *fda*::pNST260+*hlpA^Sp^-mTurquoise2-*Cm^R^ | This work |  |
| VL8175 | | HSISS4 *fda*::pNST260+*hlpA^Sp^-mNeonGreen-Cm*^R^ | This work |  |
| VL8176 | | HSISS4 *fda*::pNST260+*hlpA^Sp^-msfYFP-*Spec^R^ | This work |  |
| VL8277 | | HSISS4 *fda*::pNST260+*hlpA^Sp^-mScarlet-I-*Kan^R^ | This work |  |
| *Streptococcus pyogenes* | | |  |  |
| NV28 | | Wild-type strain *hsdR::ery* | (Bjanes et al. 2024) |  |
| VL8295 | | NV28 *fda*::pNST260+*hlpA^Sp^-mTurquoise2-*Spec^R^ | This work |  |
| VL8225 | | NV28*fda*::pNST260+*hlpA^Sp^-mNeonGreen-*Cm^R^ | This work |  |
| VL8226 | | NV28*fda*::pNST260+*hlpA^Sp^-msfYFP-*Spec^R^ | This work |  |
|  | |  |  |  |
| *Streptococcus pneumoniae* | | |  |  |
| VL1 | D39V | | (Slager et al. 2018) |  |
| VL1048 | D39V p*PEP:: P_F6_-tetR-P_T5-3_-luc-gfp* | | (Sorg et al. 2020) |  |
| VL877 | D39V *hlpA*:: *hlpA_hlpA-mNeonGreen-*Cm^R^ | | This work |  |
| VL1459 | D39V *hlpA*:: *hlpA_hlpA-mKate2-*Cm^R^ | | (Kjos and Veening 2014) |  |
| VL1634 | D39V *hlpA*:: *hlpA_hlpA-msfYFP-*Cm^R^ | | Lab collection, unpublished |  |
| VL1778 | D39V *hlpA*:: *hlpA_hlpA-mTurquoise2-*Cm^R^ | | This work |  |
| VL1780 | D39V *hlpA*:: *hlpA_hlpA-mScarlet-I-*Cm^R^ | | (Kurushima et al. 2020) |  |

**Supplementary Table S5 Overlapping and cloning PCR Subfragments**

Cloning of *hlpA-fp*’s in *S. pneumoniae* chromosome

| PCR | Primer 1 | Primer 2 |
| --- | --- | --- |
| Upstream homologous region of *hlpA* in *S. pneumoniae* | hlpA-up-F | hlpA-up-R |
| Downstream homologous region of *hlpA* in *S. pneumoniae* | cam-hlpA-down-F | cam-hlpA-down-R |
| *mTurquoise2* amplification | mTurquoise2-F | mTurquoise2-R |
| *mNeonGreen* amplification | mNeon-F | mNeon-R |

Cloning of *hlpA-fp* and antibiotic-resistant cassettes in pJet in *E. coli*

| PCR | Primer 1 | Primer 2 |
| --- | --- | --- |
| Km^R^ cassette | EcoRIKanProm_F | EcoRIK7Kanterm3 |
| Cm^R^ cassette | EcoRIK7catQ5' | HindK7catQ3' |
| Ery^R^ cassette | EcoRIEry_F | HindEry_R |
| *hlpA^Sp^-fp* amplifications to clone in pJet1.2 | hlpA_LB_F | Cm-int-R |
| Spec^R^ cassette | EcoRISpecFwdbis | HindSpecRev |

Cloning of *hlpA-fp* in *S. thermophilus hlpA* locus

| PCR | Primer 1 | Primer 2 |
| --- | --- | --- |
| Upstream homologous region (including *hlpA^St^*) of *hlpA^St^* locus (PCRhlpA_UP) | SthHlpANot_1 | hlpA-R-rbs_B |
| Downstream homologous region of *hlpA^St^* locus (PCRhlpA_DOWN) | hlpA-down-I | SthHlpA_Apa4 |
| *hlpA^Sp^-fp-*Ab^R^ amplifications to introduce a RBS (PCRhlpASp-fp-Ab^R^) | hlpA-F-rbs_C | FluoAbR2 |

Cloning of constitutively expressed *hlpA-fp* in *S. salivarius tRNA^Ser^* locus

| PCR | Primer 1 | Primer 2 |
| --- | --- | --- |
| P*_32_* at the *tRNA^Ser^* locus | UF_tRNAser | R_P32 |
| Spec^R^ cassette at the *tRNA^Ser^* locus | F_spec | DR_tRNAser2 |
| *hlpA-mKate2* amplification (for P*_32_* fusion) | F_hlpA_(P32) | R_mKate2 |
| *hlpA-mNeonGreen* amplification (for P*_32_* fusion) | F_hlpA_(P32) | R_neonGreen |
| *hlpA-mScarlet-I* amplification (for P*_32_* fusion) | F_hlpA_(P32) | R_Scarlet |
| *hlpA-mTurquoise2* amplification (for P*_32_* fusion) | F_hlpA_(P32) | R_mTurquoise |
| *hlpA-msfYFP* amplification (for P*_32_* fusion) | F_hlpA_(P32) | R_YFP |

Cloning of *hlpA-fp* and antibiotic-resistant cassettes in pNST260+ in *E. coli*

| PCR | Primer 1 | Primer 2 |
| --- | --- | --- |
| pNST260+ backbone | St1attR_For | St1Int_Rev |
| *hlpA^Sp^-fp-Ab^R^* amplifications (+RBS) | pNST_Rbs-hlpA | FluoAbR2 |

Cloning of inducible *hlpA-fp* in *S. salivarius tRNA^Ser^* locus

| PCR | Primer 1 | Primer 2 |
| --- | --- | --- |
| Upstream homologous region of *tRNA^Ser^* locus | UF_tRNAser | UR_tRNAser |
| *tetR*-P*_tet_* amplification | F_tetR_(ser) | R_tetR |
| Spec^R^ cassette at the *tRNA^Ser^* locus | F_spec | DR_tRNAser2 |
| *hlpA-mNeonGreen* at the *tRNA^Ser^* locus (for P*_tet_* fusion) | F_hlpA_(Ptet) | DR_tRNAser2 |
| *hlpA-mScarlet-I* at the *tRNA^Ser^* locus (for P*_tet_* fusion) | F_hlpA_(Ptet) | DR_tRNAser2 |
| *hlpA-mTurquoise2* at the *tRNA^Ser^* locus (for P*_tet_* fusion) | F_hlpA_(Ptet) | DR_tRNAser2 |

Cloning of inducible *ftsZ-fp* in *S. salivarius tRNA^Ser^* locus

| PCR | Primer 1 | Primer 2 |
| --- | --- | --- |
| P*_tet_* at the *tRNA^Ser^* locus | UF_tRNAser | R_tetR |
| Spec^R^ cassette at the *tRNA^Ser^* locus | F_spec | DR_tRNAser2 |
| *mNeonGreen* at the *tRNA^Ser^* locus | F_linker-fp | DR_tRNAser2 |
| *mScarlet-I* at the *tRNA^Ser^* locus | F_linker-fp | DR_tRNAser2 |
| *mTurquoise2* at the *tRNA^Ser^* locus | F_linker-fp | DR_tRNAser2 |
| *ftsZ* amplification (for P*_tet_* fusion) | F_ftsZ_(Ptet) | R_ftsZ_linkerfp |

Cloning of constitutively expressed *divIVA-fp* in *S. salivarius tRNA^Ser^* locus

| PCR | Primer 1 | Primer 2 |
| --- | --- | --- |
| P*_32_* at the *tRNA^Ser^* locus | UF_tRNAser | R_P32 |
| *mNeonGreen* at the *tRNA^Ser^* locus | F_linker-fp | DR_tRNAser2 |
| *mScarlet-I* at the *tRNA^Ser^* locus | F_linker-fp | DR_tRNAser2 |
| *mTurquoise2* at the *tRNA^Ser^* locus | F_linker-fp | DR_tRNAser2 |
| *divIVA* amplification (for P*_32_* fusion) | F_divIVA_(P32) | R_divIVA_linkerfp |

Cloning of inducible *divIVA-fp* in *S. salivarius tRNA^Ser^* locus

| PCR | Primer 1 | Primer 2 |
| --- | --- | --- |
| P*_xyl1_* at the *tRNA^Ser^* locus | UF_tRNAser | R_pZX9_ATG |
| P*_xyl2_* at the *tRNA^Ser^* locus | UF_tRNAser | R_pZX9_ATG |
| *divIVA-fp* at the *tRNA^Ser^* locus (for P*_xyl1/2_* fusion) | F_DivIVA_(Pxyl) | DR_tRNAser2 |

Cloning of inducible *ftsZ-mNeonGreen* in *S. salivarius tRNA^Thr^* locus

| PCR | Primer 1 | Primer 2 |
| --- | --- | --- |
| Upstream homologous region of *tRNA^Thr^* locus | UF_tRNAthr | UR_tRNAthr |
| Cm^R^ cassette at the *tRNA^Thr^* locus | F_cat | DR_tRNAthr |
| *tetR*-P*_tet_*-*ftsZ-mNeonGreen* amplification | F_tetR_(thr) | R_nGreen_(cat) |
| *xylR*-P*_xyl1_*-*divIVA-mScarlet-I* amplification | F_xylR_GOR | R_fp_(ery) |

Cloning of inducible *divIVA-mScarlet-I* in *S. salivarius gor* locus

| PCR | Primer 1 | Primer 2 |
| --- | --- | --- |
| Upstream homologous region of *gor* locus | Fw.Up.gor | Rev.Up.gor |
| Downstream homologous region of *gor* locus (for *ery^R^* fusion) | DF_GOR_(ery) | Rev.Dn.gor |
| Ery^R^ cassette amplification | Uplox66 | DNlox71 |
| *xylR*-P*_xyl1_*-*divIVA-mScarlet-I* amplification | F_xylR_GOR | R_fp_(ery) |

**Supplementary Table S6 Summary table for fluorescent protein constructs**

| Construct | Description |
| --- | --- |
| P*_hlpA_-hlpA-hlpA^Sp^-mTurquoise2* | Constitutive cyan signal condensed on the nucleoid |
| P*_hlpA_-hlpA-hlpA^Sp^-mNeonGreen* | Constitutive green signal condensed on the nucleoid |
| P*_hlpA_-hlpA-hlpA^Sp^-msfYFP* | Constitutive yellow signal condensed on the nucleoid |
| P*_hlpA_-hlpA-hlpA^Sp^-mScarlet-I* | Constitutive red signal condensed on the nucleoid |
| P*_hlpA_-hlpA-hlpA^Sp^-mKate2* | Constitutive red signal condensed on the nucleoid |
| P*_6_-hlpA^Sp^-mTurquoise2* | Constitutive cyan low signal condensed on the nucleoid |
| P*_6_-hlpA^SP^-mNeonGreen* | Constitutive green low signal condensed on the nucleoid |
| P*_6_-hlpA^Sp^-msfYFP* | Constitutive yellow low signal condensed on the nucleoid |
| P*_6_-hlpA^Sp^-mScarlet-I* | Constitutive red low signal condensed on the nucleoid |
| P*_32_-hlpA^Sp^-mTurquoise2* | Constitutive cyan signal condensed on the nucleoid |
| P*_32_-hlpA^Sp^-mNeonGreen* | Constitutive green signal condensed on the nucleoid |
| P*_32_-hlpA^Sp^-msfYFP* | Constitutive yellow signal condensed on the nucleoid |
| P*_32_-hlpA^Sp^-mScarlet-I* | Constitutive red signal condensed on the nucleoid |
| P*_32_-hlpA^Sp^-mKate2* | Constitutive red signal condensed on the nucleoid |
| P*_tet_-hlpA^Sp^-mTurquoise2* | Inducible cyan signal condensed on the nucleoid |
| P*_tet_-hlpA^Sp^-mNeonGreen* | Inducible green signal condensed on the nucleoid |
| P*_tet_-hlpA^Sp^-mScarlet-I* | Inducible red signal condensed on the nucleoid |
| P*_tet_- ftsZ-mTurquoise2* | Inducible cyan signal located at mid cell |
| P*_tet_-ftsZ-mNeonGreen* | Inducible green signal located at mid cell |
| P*_tet_- ftsZ-mScarlet-I* | Inducible red signal located at mid cell |
| P*_32_- divIVA-mTurquoise2* | Consitutive cyan signal located at mid cell and cell poles |
| P*_32_-divIVA-mNeonGreen* | Consitutive green signal located at mid cell and cell poles |
| P*_32_- divIVA-mScarlet-I* | Consitutive red signal located at mid cell and cell poles |
| P*_xyl1_- divIVA-mScarlet-I* | Inducible high red signal located at mid cell and cell poles |
| P*_xyl2_- divIVA-mScarlet-I* | Inducible dim red signal located at mid cell and cell poles |

Specific *S. themophilus* constructs are highlighted in grey.

ICE constructs are highlighted in red.

Specific *S. salivarius* constructs are highlighted in blue.

**Supplementary material: fusion protein DNA sequences**

*hlpA* sequence is highlighted in bold, the linker in italics and the fluorescent protein gene is underlined.

*>hlpA^Sp^-mTurquoise2*

**ATGGCAAACAAACAAGATTTGATCGCTAAAGTAGCAGAAGCTACAGAATTGACTAAGAAAGACTCAGCAGCAGCAGTTGAAGCTGTATTTGTAGCAGTAGCTGACTATCTTGCAGCTGGTGAAAAAGTTCAATTGATCGGTTTTGGTAACTTTGAAGTTCGTGAGCGTGCAGAACGTAAAGGTCGCAACCCACAAACTGGTAAAGAAATGACAATTGCAGCTTCTAAAGTACCAGCATTCAAAGCTGGTAAAGCTCTTAAAGACGCTGTTAAA***GGATCCGGATCTGGTGGAGAAGCTGAAGCTAAAGGATCA*GTGAGCAAGGGCGAGGAGCTGTTCACCGGGGTGGTGCCCATCCTGGTCGAGCTGGACGGCGACGTAAACGGCCACAAGTTCAGCGTGTCCGGCGAGGGCGAGGGCGATGCCACCTACGGCAAGCTGACCCTGAAGTTCATCTGCACCACCGGCAAGCTGCCCGTGCCCTGGCCCACCCTCGTGACCACCCTGTCCTGGGGCGTGCAGTGCTTCGCCCGCTACCCCGACCACATGAAGCAGCACGACTTCTTCAAGTCCGCCATGCCCGAAGGCTACGTCCAGGAGCGCACCATCTTCTTCAAGGACGACGGCAACTACAAGACCCGCGCCGAGGTGAAGTTCGAGGGCGACACCCTGGTGAACCGCATCGAGCTGAAGGGCATCGACTTCAAGGAGGACGGCAACATCCTGGGGCACAAGCTGGAGTACAACTACTTCAGCGACAACGTCTATATCACCGCCGACAAGCAGAAGAACGGCATCAAGGCCAACTTCAAGATCCGCCACAACATCGAGGACGGCGGCGTGCAGCTCGCCGACCACTACCAGCAGAACACCCCCATCGGCGACGGCCCCGTGCTGCTGCCCGACAACCACTACCTGAGCACCCAGTCCAAGCTGAGCAAAGACCCCAACGAGAAGCGCGATCACATGGTCCTGCTGGAGTTCGTGACCGCCGCCGGGATCACTCTCGGCATGGACGAGCTGTACAAGTAA

*>hlpA^Sp^-mNeonGreen*

**ATGGCAAACAAACAAGATTTGATCGCTAAAGTAGCAGAAGCTACAGAATTGACTAAGAAAGACTCAGCAGCAGCAGTTGAAGCTGTATTTGCAGCAGTAGCTGACTATCTTGCAGCTGGTGAAAAAGTTCAATTGATCGGTTTTGGTAACTTTGAAGTTCGTGAGCGTGCAGAACGTAAAGGTCGCAACCCACAAACTGGTAAAGAAATGACAATTGCAGCTTCTAAAGTACCAGCATTCAAAGCTGGTAAAGCTCTTAAAGACGCTGTTAAA***GGATCCGGATCTGGTGGAGAAGCTGCAGCTAAAGGATCAGTCTCT*GAAGAAGATAATATGGCTAGTTTGCCTGCTACCCACGAATTACACATTTTTGGATCAATCAATGGAGTCGATTTTGATATGGTTGGTCAAGGAACCGGTAATCCAAATGATGGATACGAAGAATTGAATCTTAAGAGTACTAAGGGTGATTTACAGTTTTCTCCATGGATTTTGGTCCCTCATATCGGATACGGTTTTCACCAATACTTGCCATATCCTGATGGAATGTCTCCTTTTCAGGCTGCAATGGTTGATGGATCAGGTTATCAAGTCCATCGTACAATGCAGTTTGAAGATGGTGCTAGTCTTACCGTTAATTATCGTTACACATATGAAGGATCTCACATTAAAGGTGAAGCCCAAGTAAAGGGAACAGGTTTTCCAGCAGATGGACCTGTTATGACTAATTCACTTACCGCCGCTGATTGGTGTCGTAGTAAAAAGACTTATCCAAATGATAAGACAATCATCTCTACTTTTAAGTGGTCATACACAACTGGAAATGGTAAACGTTATCGTTCAACTGCTCGTACCACATACACCTTTGCAAAACCAATGGCAGCCAATTACCTTAAGAATCAACCTATGTACGTCTTTCGTAAGACAGAACTTAAGCATTCTAAGACTGAACTTAATTTTAAGGAATGGCAGAAGGCTTTTACTGATGTAATGGGTATGGATGAATTGTATAAATAA

*>hlpA^Sp^-msfYFP*

**ATGGCAAACAAACAAGATTTGATCGCTAAAGTAGCAGAAGCTACAGAATTGACTAAGAAAGACTCAGCAGCAGCAGTTGAAGCTGTATTTGCAGCAGTAGCTGACTATCTTGCAGCTGGTGAAAAAGTTCAATTGATCGGTTTTGGTAACTTTGAAGTTCGTGAGCGTGCAGAACGTAAAGGTCGCAACCCACAAACTGGTAAAGAAATGACAATTGCAGCTTCTAAAGTACCAGCATTCAAAGCTGGTAAAGCTCTTAAAGACGCTGTTAAA***GGATCCGGATCTGGTGGAGAAGCTGCAGCTAAATCTAGTTCTAGAGGATCTGGTGGAGAAGCTGCAGCTAAAGCTGGA*TCAAAAGGAGAAGAACTTTTTACAGGTGTAGTACCTATCTTGGTTGAATTGGATGGTGATGTTAACGGTCACAAATTTTCTGTACGTGGTGAAGGTGAAGGTGATGCAACTAACGGTAAATTGACACTTAAATTGATTTGTACAACTGGAAAACTTCCTGTTCCTTGGCCTACTCTTGTTACAACATTGGGTTATGGACTTATGTGTTTTGCTCGTTATCCTGATCATATGAAACGTCACGATTTTTTTAAATCTGCTATGCCAGAAGGTTATGTACAAGAACGTACAATTTCATTTAAAGATGACGGAACATATAAAACACGTGCTGAAGTAAAATTCGAAGGTGACACTCTTGTTAATCGTATCGAATTGAAAGGAATCGATTTCAAAGAAGATGGTAACATTTTGGGACACAAACTTGAATACAACTTCAACTCTCATAATGTTTATATCACAGCTGACAAACAAAAAAACGGTATTAAAGCTAATTTTAAAATTCGTCACAATGTTGAAGATGGAGGAGTTCAATTGGCTGATCATTATCAACAAAATACACCAATCGGAGACGGACCAGTATTGCTTCCAGATAACCACTACCTTTCTTATCAATCAAAACTTTCAAAAGATCCTAACGAAAAACGTGACCATATGGTACTTCTTGAATTTGTTACAGCAGCAGGTATCACTCACGGTATGGACGAACTTTATAAATAA

>*hlpA^Sp^-mScarlet-I*

**ATGGCAAACAAACAAGATTTGATCGCTAAAGTAGCAGAAGCTACAGAATTGACTAAGAAAGACTCAGCAGCAGCAGTTGAAGCTGTATTTGCAGCAGTAGCTGACTATCTTGCAGCTGGTGAAAAAGTTCAATTGATCGGTTTTGGTAACTTTGAAGTTCGTGAGCGTGCAGAACGTAAAGGTCGCAACCCACAAACTGGTAAAGAAATGACAATTGCAGCTTCTAAAGTACCAGCATTCAAAGCTGGTAAAGCTCTTAAAGACGCTGTTAAA***GGATCCGGATCTGGTGGAGAAGCTGCAGCTAAAGGATCA*GTGAGTAAAGGCGAAGCTGTAATAAAGGAGTTCATGAGATTTAAAGTACACATGGAGGGGTCAATGAACGGTCATGAATTTGAAATTGAGGGAGAGGGAGAGGGAAGACCGTATGAGGGTACGCAAACCGCCAAATTGAAGGTAACGAAGGGGGGACCTCTTCCTTTTTCATGGGACATTCTTAGCCCGCAGTTCATGTATGGGAGCCGAGCATTTATTAAGCACCCAGCGGACATACCAGATTATTACAAACAATCTTTCCCTGAAGGTTTCAAGTGGGAGAGAGTTATGAACTTTGAGGACGGAGGAGCTGTAACAGTGACCCAAGATACTTCACTAGAGGACGGGACTCTTATTTACAAAGTCAAACTACGAGGAACGAACTTTCCGCCTGACGGCCCTGTTATGCAGAAAAAGACTATGGGCTGGGAAGCCTCTACGGAGCGCCTATACCCAGAGGATGGTGTTTTGAAAGGTGACATTAAAATGGCACTTCGCCTAAAGGACGGAGGCCGATACCTAGCAGATTTTAAAACAACGTATAAAGCTAAGAAACCTGTACAAATGCCAGGAGCATATAATGTTGATCGAAAATTAGATATTACTTCTCATAACGAGGACTACACAGTGGTTGAACAATATGAACGTAGCGAAGGTCGTCATTCAACTGGGGGCATGGACGAACTATATAAATAA

>*hlpA^Sp^-mKate2*

**ATGGCAAACAAACAAGATTTGATCGCTAAAGTAGCAGAAGCTACAGAATTGACTAAGAAAGACTCAGCAGCAGCAGTTGAAGCTGTATTTGCAGCAGTAGCTGACTATCTTGCAGCTGGTGAAAAAGTTCAATTGATCGGTTTTGGTAACTTTGAAGTTCGTGAGCGTGCAGAACGTAAAGGTCGCAACCCACAAACTGGTAAAGAAATGACAATTGCAGCTTCTAAAGTACCAGCATTCAAAGCTGGTAAAGCTCTTAAAGACGCTGTTAAA***GGATCCGGATCTGGTGGAGAAGCTGCAGCTAAAGGATCA*GAACTTATCAAGGAAAATATGCACATGAAATTGTACATGGAAGGAACAGTAAATAATCACCACTTTAAATGTACCTCAGAAGGAGAAGGAAAACCATATGAAGGTACTCAAACCATGCGTATTAAGGCCGTTGAAGGTGGACCATTGCCTTTTGCCTTTGATATTCTTGCTACATCTTTTATGTACGGATCAAAGACTTTTATCAATCATACCCAAGGTATCCCAGATTTCTTTAAACAGTCATTTCCTGAAGGATTTACATGGGAACGTGTCACAACTTATGAAGATGGTGGAGTATTGACAGCAACTCAAGATACATCTTTGCAAGATGGTTGTCTTATCTACAATGTAAAGATCCGTGGAGTTAATTTTCCAAGTAATGGTCCTGTTATGCAGAAAAAGACCCTTGGATGGGAAGCATCTACCGAAACATTATATCCTGCCGATGGTGGATTGGAAGGTCGTGCTGATATGGCATTGAAACTTGTCGGTGGAGGTCACCTTATCTGTAATTTGAAGACCACATACCGTTCTAAAAAGCCAGCTAAGAATCTTAAGATGCCTGGTGTTTACTACGTCGATCGTCGTTTAGAACGTATCAAAGAAGCAGATAAGGAAACTTATGTTGAACAGCACGAAGTAGCCGTCGCACGTTATTGTGATTTGCCTAGTAAATTGGGACACCGTTAA

**References**

Bellanger X, Roberts AP, Morel C et al. Conjugative transfer of the integrative conjugative elements ICESt1 and ICESt3 from *Streptococcus thermophilus*. *J Bacteriol* 2009;**191**:2764-2775. <https://doi.org/10.1128/JB.01412-08>

Bjanes E, Stream A, Janssen AB et al. An efficient in vivo-inducible CRISPR interference system for group A Streptococcus genetic analysis and pathogenesis studies. *mBio* 2024;**15**:e0084024. <https://doi.org/10.1128/mbio.00840-24>

Kjos M, Veening JW. Tracking of chromosome dynamics in live *Streptococcus pneumoniae* reveals that transcription promotes chromosome segregation. *Mol Microbiol* 2014;**91**:1088-1105. <https://doi.org/10.1111/mmi.12517>

Kurushima J, Campo N, van Raaphorst R et al. Unbiased homeologous recombination during pneumococcal transformation allows for multiple chromosomal integration events. *Elife* 2020;**9**. <https://doi.org/10.7554/eLife.58771>

Mignolet J, Cerckel G, Damoczi J et al. Subtle selectivity in a pheromone sensor triumvirate desynchronizes competence and predation in a human gut commensal. *Elife* 2019;**8**. <https://doi.org/10.7554/eLife.47139>

Mignolet J, Fontaine L, Sass A et al. Circuitry Rewiring Directly Couples Competence to Predation in the Gut Dweller *Streptococcus salivarius*. *Cell Rep* 2018;**22**:1627-1638. <https://doi.org/10.1016/j.celrep.2018.01.055>

Slager J ,Aprianto R, Veening JW. Deep genome annotation of the opportunistic human pathogen *Streptococcus pneumoniae* D39. *Nucleic Acids Res* 2018;**46**:9971-9989. <https://doi.org/10.1093/nar/gky725>

Sorg RA, Gallay C, Van Maele L et al. Synthetic gene-regulatory networks in the opportunistic human pathogen *Streptococcus pneumoniae*. *Proc Natl Acad Sci U S A* 2020;**117**:27608-27619. <https://doi.org/10.1073/pnas.1920015117>

Van den Bogert B, Boekhorst J, Herrmann R et al. Comparative genomics analysis of *Streptococcus* isolates from the human small intestine reveals their adaptation to a highly dynamic ecosystem. *PLoS One* 2014;**8**:e83418. <https://doi.org/10.1371/journal.pone.0083418>
